# Bioenergetic profiles and respiratory control in mitochondrial physiology: Precision analysis of oxidative phosphorylation

**DOI:** 10.1113/EP092792

**Published:** 2025-08-01

**Authors:** Alba Timón‐Gómez, Carolina Doerrier, Zuzana Sumbalová, Luiz F. Garcia‐Souza, Eleonora Baglivo, Luiza H. D. Cardoso, Erich Gnaiger

**Affiliations:** ^1^ Oroboros Instruments Innsbruck Austria

**Keywords:** coupling control, electron transfer system (ETS), high‐resolution respirometry (HRR), OXPHOS, pathway control, substrate‐uncoupler‐inhibitor‐titration (SUIT) protocol

## Abstract

Oxidative phosphorylation (OXPHOS) is fundamental to mitochondrial function. Respirometry with living cells provides limited information compared to precision OXPHOS analysis with mitochondrial preparations, including isolated mitochondria, tissue homogenates, permeabilized tissues, and permeabilized cells. We studied mouse mitochondria from brain, a glucose‐dependent tissue, and from heart, which relies highly on fatty acid oxidation (FAO). HEK 293T cells were analysed as a widely used experimental model. Human peripheral blood mononuclear cells (PBMCs) and platelets were obtained from non‐invasive liquid biopsies, considering their potential as mitochondrial biomarkers. Twenty respiratory states were interrogated applying two substrate–uncoupler–inhibitor titration (SUIT) reference protocols in parallel. Convergent electron transfer (ET) into the coenzyme Q junction increased OXPHOS and ET capacities compared to separately stimulated pathways. In mouse heart and human PBMCs, OXPHOS capacities were identical to ET capacities in every pathway state. While this equivalence applied to the NADH‐linked pathway in platelets, ET capacity exceeded OXPHOS capacity supported by NADH‐linked substrates plus succinate. Surprisingly, mouse brain exhibited the highest excess ET capacity in the NADH‐linked pathway. In contrast, ET capacity of different batches of HEK 293T cells varied at constant OXPHOS capacity. Precision OXPHOS analysis enables attribution of respiratory performance to nutrient‐specific pathways. In studies ranging from exercise physiology to mitochondrial diseases, metabolic adjustments must be distinguished from functional defects. Bioenergetic profiles obtained by precision OXPHOS analysis gain perspective in the context of comparative mitochondrial physiology.

## Abstract (highlights)

- **What is the central question of this study?**
  OXPHOS is fundamental for mitochondrial function and cellular metabolism: can the physiological relevance of mitochondrial function be effectively addressed using precision OXPHOS analysis?
- **What is the main finding and its importance?**
  Bioenergetic profiles revealed mitochondrial functional diversity and tissue‐ and cell‐specific metabolic patterns. Precision OXPHOS analysis is essential for understanding mitochondrial function, formulating robust working hypotheses, selecting appropriate models, and explaining tissue‐specific differences. Bioenergetic profiling unravels the complexities of mitochondrial respiratory control on the basis of a consistent comparative database.

## 1 INTRODUCTION

Mitochondria contain the machinery for producing ATP through oxidative phosphorylation (OXPHOS) in most eukaryotic cells. OXPHOS has a central position in cellular metabolism, impacting cellular homeostasis. Comparable to the ergorespirometric ${\dot{V}}_{O_{2}\max}$ of the whole organism (the maximal capacity of oxygen consumption at stepwise increased workload), mitochondrial ‘cell ergometry’ yields a measure of maximum ADP‐stimulated OXPHOS capacity (${J_{O_{2}}}_{\max}$) (Gnaiger, 2009; Rasmussen et al., 2001). Determination of OXPHOS capacity is not possible in respirometric studies of living cells but requires mitochondrial preparations: the barrier function of the plasma membrane is disrupted to obtain ${J_{O_{2}}}_{\max}$ of well‐coupled mitochondria stimulated by saturating concentrations of ADP and inorganic phosphate. However, to reconstitute tricarboxylic acid (TCA) cycle function, mitochondrial preparations must be supplied with complex substrate combinations (Gnaiger, 2009; Rasmussen et al., 2001) to avoid underestimation of ${J_{O_{2}}}_{\max}$ in isolated mitochondria (Villani et al., 1998). OXPHOS capacity, together with oxygen supply to the muscle, determines ${\dot{V}}_{O_{2}\max}$ (Gifford et al., 2016; Hepple et al., 2002). Tissue‐specific alterations of OXPHOS capacity are related to many pathophysiological conditions (Balmaceda et al., 2024), including metabolic, cardiovascular, neuromuscular and neurodegenerative disorders, ageing (reviewed in Nunnari & Suomalainen, 2012; Wallace, 2011), and several types of cancer. Thus, evaluation of mitochondrial function plays an important role in comparative physiology, sport science, and preventive and therapeutic medicine including innovative mitochondrial transplantation therapy (Jiao et al., 2024). Precision OXPHOS analysis beyond the scope of respirometric protocols restricted to living cells enhances understanding the mechanisms of energy transformation in health and disease.

The interest in OXPHOS analysis is fuelled by a growing awareness of the contribution of mitochondrial metabolism to the aetiology of many diseases and by technological developments that allow robust examination. High‐resolution respirometry (HRR) measures mitochondrial respiration in a sensitive and reproducible manner (Gnaiger et al., 1995; Gnaiger, 2001). Analysis of coupling and pathway control is performed using substrate–uncoupler–inhibitor titration (SUIT) protocols with sequential titrations for real‐time OXPHOS analysis in mitochondrial preparations (Gnaiger, 2009, 2020). The evaluation of multiple respiratory states sets a benchmark in the context of precision OXPHOS analysis, revealing the diversity of mitochondrial respiratory control in different cell types, tissues and species.

The SUIT reference protocols RP1 and RP2 interrogate 20 mitochondrial pathway and coupling control states (Doerrier et al., 2018). Applications of these SUIT protocols have been published with short conceptual explanations (Doerrier et al., 2018; Jang et al., 2018). Here we provide an in‐depth analysis of each titration step to rationalize results from the complex reference protocols and similar shorter SUIT protocols. Respiratory rates may differ when the same substrate combinations are obtained after different sequences of substrate titrations resulting in comparable respiratory states (Votion et al., 2012). We analysed whether comparable states from RP1 and RP2, achieved through different titration sequences, result in similar respiratory fluxes.

We address the physiological relevance of mitochondrial functional diversity. For the first time, RP1 and RP2 were applied to compare respiratory control patterns across various species, tissues and cell types. We studied mouse mitochondria from brain as a highly glucose‐dependent tissue, and from heart, which is more dependent on fatty acid oxidation (FAO). The analysis of the established metabolic differences between brain and heart was used as a proof‐of‐concept of the proper design of RP1 and RP2 to provide specific bioenergetic profiles. Peripheral blood mononuclear cells (PBMCs) and platelets were obtained from human liquid biopsies, considering their role as potential mitochondrial biomarkers for diagnostic purposes. We hypothesized that these two cell lines might show similar bioenergetic profiles due to their similar environmental conditions (Oemer et al., 2018). Cultured cell lines, although frequently used as models to study mitochondrial (dys)function, have previously been shown to be limited in representing the bioenergetic properties of a tissue (Schöpf et al., 2016). Thus, the human embryonic kidney (HEK) 293T cell line was analysed as a widely used experimental model, and its bioenergetic profile was compared against the profiles of the above tissues and cell lines to detect potential correlations of mitochondrial function.

Bioenergetic profiles revealed distinct tissue‐specific metabolic patterns across all models. These findings highlight the value of precision OXPHOS analysis to provide detailed information on coupling and pathway control (see ‘Theoretical background’) to: (1) quantify physiologically relevant maximum OXPHOS capacities; (2) extend our knowledge of the diversity and functional significance of bioenergetic profiles on the basis of a consistent comparative database; (3) select the most suitable model for addressing specific research questions (e.g., drug testing); and (4) improve our understanding of respiratory control required to formulate robust working hypotheses that can distinguish between functional defects and adjustments to altered metabolic demands.

### 1.1 Theoretical background

**FIGURE 1:**
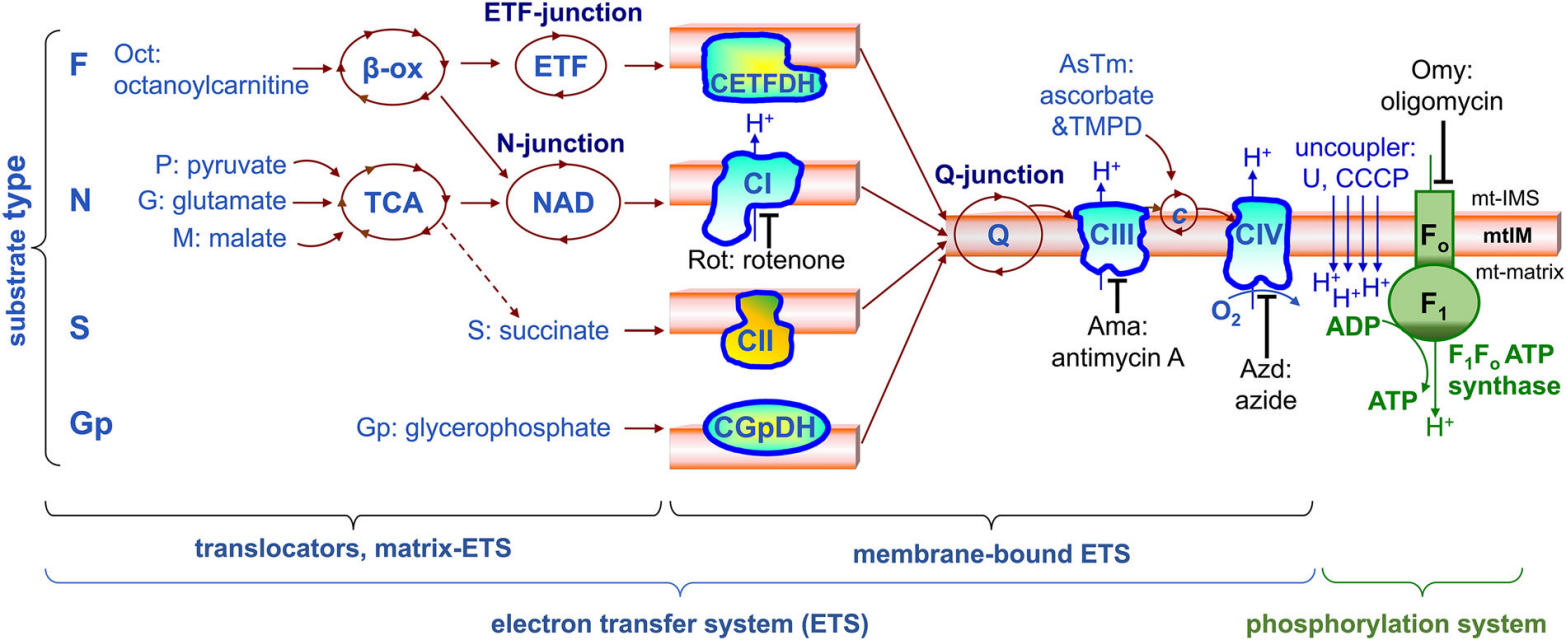
Convergent ET and OXPHOS. ET converges at the N‐, ETF‐ and Q‐junctions. Pathways (F, N, S, Gp) are named according to the substrate type. See Table 1 for further abbreviations. Modified after Gnaiger (2020, 2024). CETFDH, Complex of ETF dehydrogenase; CGpDH, Complex of glycerophosphate dehydrogenase; CI–IV, Complexes I–IV; ET, electron transfer; ETF, electron‐transferring flavoprotein; mt‐IMS, mt‐intermembrane space; NAD, NADH + NAD^+^; Q, UQ+UQH_2_; TCA, tricarboxylic acid; β‐ox, beta‐oxidation.

In OXPHOS, endergonic (uphill) ATP synthesis is coupled to exergonic (downhill) H^+^‐linked electron transfer (ET) from reduced substrates to oxygen. The electron transfer system (ETS) consists of mitochondrial matrix and membrane‐bound convergent sections (Figure 1). Reduced metabolites are transported into the mitochondrial matrix space and are oxidized in several catabolic steps. These are catalysed by enzymes of the TCA cycle and β‐oxidation cycle, and several dehydrogenases localized in the mitochondrial matrix. Electrons from NADH + H^+^ and succinate are transferred to the mitochondrial inner membrane‐bound respiratory Complexes I and II (CI and CII, respectively), and from electron‐transferring flavoprotein (ETF) to another respiratory Complex, ETF dehydrogenase (CETFDH; Gnaiger, 2024). These and additional electron entries – such as glycerophosphate dehydrogenase, dihydroorotate dehydrogenase, proline dehydrogenase, choline dehydrogenase and sulfide‐ubiquinone oxidoreductase – converge at the Q‐junction (Barrett & Dwason, 1975; Brito et al., 2009; Froman & Kennedy, 1975; Gnaiger, 2020; Hatefi et al., 1962; Ringler & Singer, 1959). Electrons are further transferred through Complex III (CIII) and cytochrome *c* to Complex IV (CIV), where oxygen is finally reduced to H_2_O (Figure 1). During this process, CI, CIII and CIV pump H^+^ across the mitochondrial inner membrane (mtIM), generating a chemiosmotic pressure difference used by the F_1_F_O_‐ATPsynthase to phosphorylate ADP to ATP. The phosphorylation system comprises ATP‐synthase, adenine nucleotide translocase and the inorganic phosphate transporter.

## 2 METHODS

### 2.1 Ethical approval

#### 2.1.1 Human samples

Six middle‐age (45–60 years old) healthy participants (3 males, 3 females) from Western Europe (Caucasians) were recruited. Written informed consent was obtained from all participants involved in the study. The study was approved by the Institutional Review Board of the Department of Sport Science (41/2016, University of Innsbruck) and conducted according to the principles expressed in the *Declaration of Helsinki*, except for registration in a database.

#### 2.1.2 Animal samples

Wild‐type C57BL/6N mice (*N* = 7, 4 males and 3 females, RRID:MGI:2159965), provided by Charles River (Cologne, Germany), were housed in clear plastic cages, and maintained at the Central Laboratory Animal Facility of the Medical University Innsbruck in a specific pathogen‐free barrier zone with constant temperature (22 ± 1°C) and 40–60 % relative humidity with 12 h light–dark cycle (07.00–19.00 h, light period). The mice were fed standard chow ad libitum and had free access to water. All mice were used at 3 months of age. Experiments on mice were conducted in accordance with the University of Innsbruck's Ethical Committee and the European Convention for the Protection of Vertebrate Animals used for Experimental and Other Scientific Purposes (CETS no. 123).

Beef heart and liver were obtained from the same four slaughterhouse cows (following the Three Rs principles – Replacement, Reduction, Refinement – in the Animal Experimentation Act 2012). Provisions stated in the European Council Directive 98/58/EC, Council Directive 2008/119/EC, Council Directive EC 1/2005, Council Directive EC No. 1099/2009, and regulations for farming animals in the Animal Welfare Act 2004 and First Regulation on Keeping Animals 2004 (Government of Austria) were followed in these animals to ensure their welfare.

### 2.2 Chemicals

The relaxing and biopsy preservation solution BIOPS (CaK_2_EGTA 2.77 mM, K_2_EGTA 7.23 mM, Na_2_ATP 5.77 mM, MgCl_2_∙6H_2_O 6.56 mM, taurine 20 mM, Na_2_‐phosphocreatine 15 mM, imidazole 20 mM, dithiothreitol 0.5 mM, MES hydrate 50 mM, pH 7.1) was used for tissue storage. Isolation buffer A (mannitol 225 mM, sucrose 75 mM, EGTA 1 mM, BSA essentially fatty acid‐free 2.5 mg mL^−1^) and B (isolation buffer A with subtilisin 0.5 g L^−1^) were used for preparation of isolated cardiac mitochondria. The following chemicals were used for cell culture: Dulbecco's modified Eagle's medium (DMEM‐high glucose; Lonza, Basel, Switzerland; LONBE12‐614F, 500 mL), glutamine (Lonza, LONBE17‐605E, 100 mL), fetal bovine serum (FBS; Gibco 10500, 500 mL, Thermo Fisher Scientific, Grand Island, NY USA), penicillin (50 U mL^−1^, Sigma‐Aldrich, Saint Louis, MO USA, P4458), streptomycin (50 µg mL^−1^, Corning, Corning, NY, USA), Dulbecco's phosphate‐buffered saline (DPBS; Lonza, LONBE17‐512F, 500 mL), trypsin/EDTA (Lonza, LONBE17‐161E, 100 mL), dimethyl sulfoxide (DMSO; Sigma‐Aldrich, 276855, 100 mL), and Trypan Blue (0.4 %, Thermo Fisher Scientific, T10282). During PBMC and platelet isolation, EDTA (Sigma‐Aldrich, 324503), Ficoll‐Paque (GE Healthcare, Cytiva, Uppsala, Sweden, GE17‐1440‐02, 100 mL), and EGTA (Sigma‐Adrich, 324626) were used.

Mitochondrial respiration medium MiR05, prepared from MiR05‐Kit (Oroboros Instruments, Innsbruck, Austria, product ID 60101‐01: EGTA 0.5 mM, MgCl_2_∙6H_2_O 3 mM, taurine 20 mM, KH_2_PO_4_ 10 mM, HEPES 20 mM, d‐sucrose 110 mM, BSA fatty acid‐free 1 g L^−1^, and lactobionic acid 60 mM, pH 7.1), was used for preparation of brain homogenate. MiR05, MiR06 (MiR05 plus catalase 280 U mL^−1^) and MiR06Cr (MiR06 with creatine 3 mg mL^−1^) were employed for HRR measurements (Baglivo et al., 2024; Doerrier et al., 2024).

The following chemicals were used in SUIT protocols (abbreviations, concentrations of stock solutions, and catalogue numbers in parentheses). Sigma‐Aldrich: cytochrome *c* (c, 4 mM; C7752); glutamate (G, 2 M; G1626), malate (M, 50 and 400 mM; M1000), pyruvate (P, 2 M; P2256), succinate (S, 1 M; S2378), carbonyl cyanide *m*‐chlorophenyl hydrazone CCCP (U, 1 mM; C2759), antimycin A (Ama, 5 mM; A8674), rotenone (Rot, 1 mM; R8875), digitonin (Dig, 10 mg mL^−1^; D5628), ascorbate (As, 800 mM; A7631), *N,N,N',N'*‐tetramethyl‐*p*‐phenylenediamine dihydrochloride (TMPD) (Tm, 200 mM; T3134), sodium azide (Azd, 4 M; S2002). Merck, Darmstadt, Germany: ADP (D, 500 mM; 117105). Santa Cruz Biotechnology (Dallas, TX, USA): glycerophosphate (Gp, 1 M; sc‐215789). Biotrend (APExBIO Technology, Houston, TX USA): octanoylcarnitine (Oct, 100 mM; B6371). Stock solutions were titrated with Hamilton microsyringes into the Oroboros chambers, to obtain the experimental concentrations indicated in figure legends.

### 2.3 Sample preparation

#### 2.3.1 Culture and cryopreservation of HEK 293T

HEK 293T cells (ATCC CRL‐3216, RRID:CVCL_0063) were obtained from the American Type Culture Collection through LGC Standards (Wesel, Germany). Cells were cultured in 10‐cm culture dishes and maintained at 37°C, in 5 % CO_2_ and 98 % humidity in DMEM, supplemented with 2 mM glutamine, 10 % FBS, and 1 % penicillin and streptomycin. Cells were passaged at 70 % confluence and were expanded starting with passage 3 and harvested at passage 6. For passaging, cells were washed with DPBS, trypsinized, and re‐seeded at a ratio of 1:6.

For final collection of cells for cryopreservation, cells from up to 50 parallel cultures were pooled in two 50‐mL Falcon tubes, spun down at 125 *g* for 5 min at room temperature (RT), and the pellets were resuspended in cryopreservation medium consisting of 90 % FBS and 10 % DMSO (https://wiki.oroboros.at/index.php/MiPNet21.14_Reference_sample_HRR; accessed 4 March 2025). Cells were counted in the presence of Trypan Blue using the Countess II automated cell counter (Thermo Fisher Scientific), diluted to a cell concentration of 60·10^6^ x/mL, transferred to cryotubes in 250 µL aliquots, and immediately frozen at −80°C. For HRR measurements, seven different batches of HEK 293T cells were used. Cells were thawed for 1 min at 37°C after 2–4 weeks of cryopreservation, resuspended in 750 µL MiR05, and counted; 2·10^6^ cells were added to each chamber, and data were normalized by cell concentration.

#### 2.3.2 Isolation of human PBMCs and platelets

Eighteen millilitres of venous blood was extracted from each participant to isolate PBMCs and platelets. Participants were fasting for 8–12 h and were advised to refrain from intensive physical activity 48 h prior to the blood draw. Blood was collected in BD Vacutainer tubes (BD, Franklin Lakes, NJ, USA) containing EDTA. PBMCs and platelets were isolated within 2 h of storage of the whole blood at RT. After sample collection, the whole blood was transferred slowly and carefully onto the top of the polyethylene barrier of a Leucosep tube (Greiner bio‐one, Unilab, Innsbruck, Austria) containing a density gradient medium (Ficoll‐Paque) and diluted with DPBS (1:1). All solutions and isolation steps were maintained and performed at RT. Afterwards, the tube was centrifuged at 1000 *g* for 10 min (centrifuge brakes were off to avoid disruption of the gradient layers) and different layers were observed: (1) plasma; (2) a fraction with PBMCs and platelets; (3) density gradient medium; (4) erythrocytes and granulocytes. The layer with plasma was kept, and the layer enriched with PBMCs and platelets was collected carefully and added into a 50‐mL Falcon tube. DPBS was added up to 25 mL, and it was centrifuged at 120 *g* for 10 min (maximum acceleration/deceleration speed 9, brake 6), generating a pellet with PBMCs and a supernatant (supernatant 1) for platelet isolation. The pellet was resuspended with 25 mL DPBS, and the tube was centrifuged at 120 *g* for 10 min (acceleration/deceleration speed 9, brake 6). The final pellet, which contains the cell fraction with PBMCs, was resuspended in 0.5 mL DPBS.

For platelet isolation, supernatant 1 was mixed with 5 mL of the clear plasma fraction and 10 mM EGTA, to prevent platelet activation and aggregation. The mix was centrifuged at 1000 *g* for 10 min (acceleration/deceleration speed 9, brake 2). The resultant pellet was gently resuspended in DPBS and 10 mM EGTA, prior to another centrifugation at 1000 *g* for 5 min (acceleration/deceleration speed 9, brake 2). The platelet fraction in the pellet was resuspended in 0.5 mL DPBS with 10 mM EGTA.

Both cell suspensions were counted twice using the Sysmex XN‐350 haematology analyser (Sysmex Corp., Kobe, Japan). The average cell concentration was used for data normalization. 5·10^6^ to 6·10^6^ PBMCs and 200·10^6^ to 260·10^6^ platelets were added immediately after isolation to each 2‐mL Oroboros chamber containing respiration medium MiR05. The suspension of isolated mitochondria in MiR05 as respiration medium leads to higher ET capacity and lower platelet activation compared to other buffers (Siewiera et al., 2021).

#### 2.3.3 Isolation of mouse cardiac mitochondria

After killing the mice by cervical dislocation, the tissues (heart and brain) were immediately removed, transferred into ice‐cold preservation solution (BIOPS), and kept on ice until their processing on the same day. Hearts were transferred to a Petri dish on ice to remove connective tissue and blood vessels. The heart tissue was washed in ice‐cold BIOPS, and the wet mass was measured. Then, the tissue was minced, suspended in isolation buffer B (1:20 w/v), and homogenized at 800 rotations per minute (rpm) with a Teflon pestle (Stuart Scientific, Staffordshire, United Kingdom, model SS2). The homogenate was diluted with isolation buffer B (1:50 w/v) and centrifuged at 800 *g* for 10 min (4°C). The resulting supernatant was centrifuged at 10000 *g* for 10 min (4°C). The pellet was carefully resuspended in isolation buffer A (1:5 w/v) and centrifuged again at 10 000 *g* for 10 min (4°C). The resulting mitochondrial pellet was resuspended in isolation buffer A (1:2 w/v), and the mitochondrial suspension was kept on ice until respiratory measurement. A 100‐µL subsample was taken and stored at −20°C for subsequent protein determination (after Lowry et al., 1951). All steps were performed on ice to preserve mitochondrial function.

#### 2.3.4 Mouse brain tissue homogenate

Brain tissue (see Section 2.3.3) was washed in ice‐cold BIOPS, and the wet mass was determined. Then, the tissue was gently homogenized using a Teflon pestle with 10 strokes at 1000 rpm in MiR05. All steps were performed on ice.

#### 2.3.5 Beef heart and liver homogenate

Beef heart and liver were obtained immediately after the killing of the animal and were placed immediately in ice‐cold BIOPS solution and transported to the laboratory on ice. Samples were minced into small pieces in ice‐cold BIOPS on the day of collection; 10 to 20 mg wet mass was recovered per sample. Tissue homogenization was performed using the PBI‐Shredder (Oroboros Instruments) in ice‐cold respiratory medium MiR05 using a custom‐made metal plate (Gross et al., 2011 and https://wiki.oroboros.at/index.php/MiPNet17.15_PBI‐Shredder_Mouse‐heart‐brain‐liver, accessed 4 March 2025).

#### 2.3.6 Induction of cytochrome *c* effect in animal and cellular models

As a model of high damage of the mitochondrial outer membrane (mtOM), beef liver and heart were homogenized with a custom‐made metal plate using the PBI‐Shredder, as described above. A 5‐fold digitonin concentration was used to induce a controlled damage of the mtOM of the HEK 293T cells inside the Oroboros chamber.

### 2.4 High‐resolution respirometry

**FIGURE 2:**
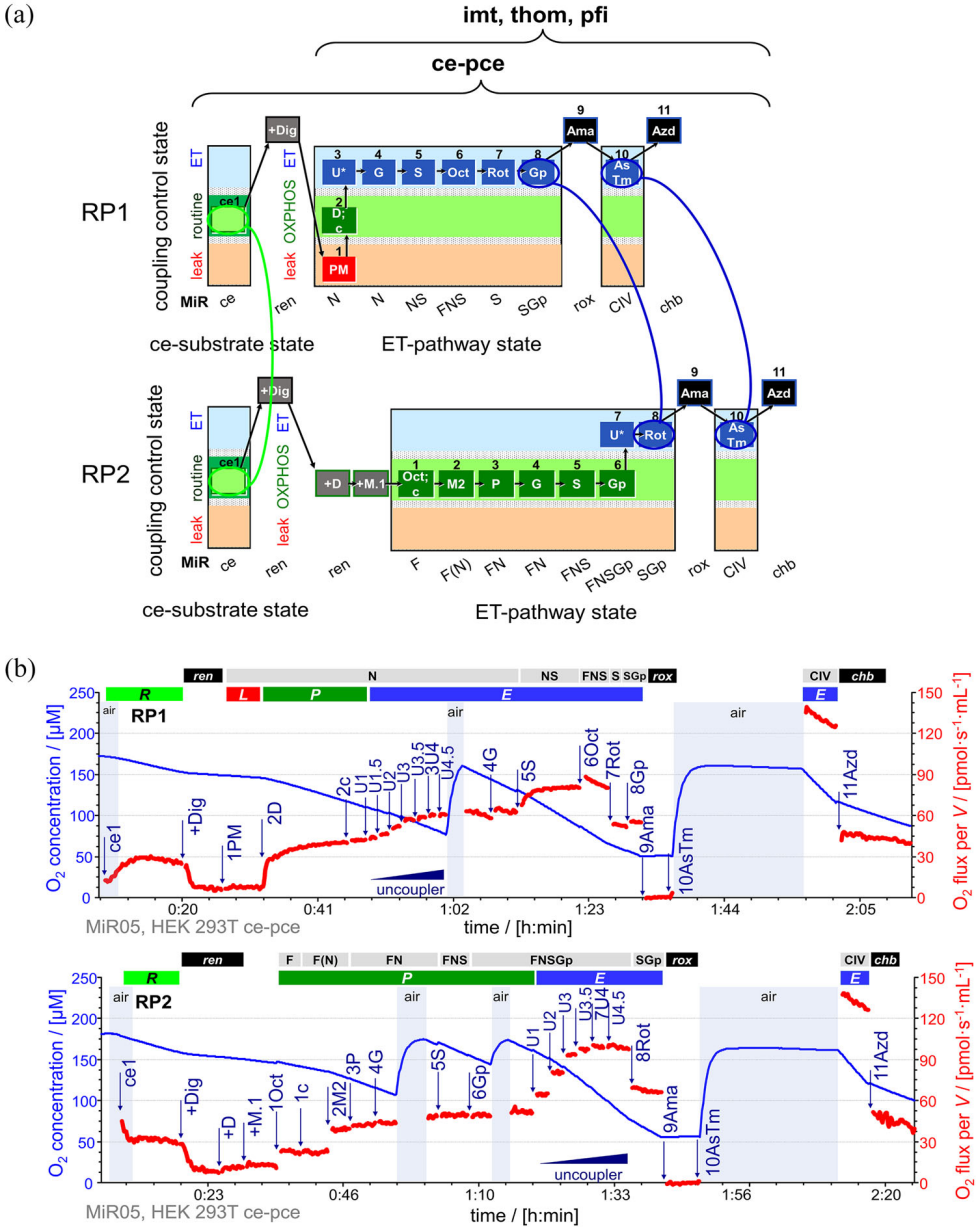
SUIT reference protocols RP1 and RP2. (a) Coupling and pathway control diagrams for mitochondrial preparations (Table 2). Comparable states in RP1 and RP2 are indicated by circles and connected by lines. Nomenclature for ET‐pathway states from Figure 1. Preparatory titration steps are indicated by ‘+’ before the added compound. Changes in the coupling control states (Table 3) and pathway control states (Table 4) are shown by sequential numbers. (b) Representative traces of O_2_ concentration (µM) (blue line) and O_2_ flux per volume *V* (pmol s^−1^ mL^−1^) (red line; corrected for instrumental background O_2_ flux) in HEK 293T cells. Titrations are indicated by arrows. Titration spikes were eliminated. Oxygen concentration was kept above 50 µM by intermittent reoxygenations (air, open chamber, blue shade indicating sections when O_2_ flux was not measured). Sequence of respiratory states in RP1, characterized by titrations and corresponding rates (see Tables 3 and 4): ce1, routine respiration *R* of living cells. +Dig, digitonin 10 mg mL^−1^; permeabilization of plasma membrane (transition from ce to pce), residual endogenous respiration *ren*. 1PM, pyruvate 5 mM and malate 2 mM; N‐pathway leak respiration N{PM}*_L_*. 2D, ADP 2.5 mM; N‐pathway OXPHOS capacity N{PM}*_P_*. 2c, cytochrome *c* 10 µM; N{PM}[c]_*P*_; test of mtOM integrity. 3U4, uncoupler titrations to optimum CCCP concentration 4 µM; NADH‐linked ET capacity N{PM}*_E_*. 4G, glutamate 10 mM; N*_E_*. 5S, succinate 10 mM; NS‐pathway ET capacity NS*_E_*. 6Oct, octanoylcarnitine 0.5 mM; FNS‐pathway ET capacity FNS*_E_*. 7Rot, rotenone 0.5 mM inhibiting CI; succinate‐pathway ET capacity S*_E_*. 8Gp, glycerophosphate 10 mM; SGp*_E_*. 9Ama, antimycin A 2.5 µM inhibiting CIII; residual oxygen consumption *rox*. 10AsTm, ascorbate 2 mM and TMPD 0.5 mM; substrates for CIV stimulation; the chamber was kept open for 20 min to allow for redox equilibration without depletion of oxygen (Doerrier et al., 2018). 11Azd, sodium azide 200 mM inhibiting CIV; oxygen‐dependent chemical background flux *chb*. Sequence of respiratory states in RP2: ce1, *R*. +Dig, *ren*. +D, stimulating *ren*. +M.1, 1Oct and 1c, malate 0.1 mM, Oct and cytochrome *c*, F‐pathway OXPHOS capacity F*_P_* = *J*(1Oct(*c*)) − *J*(+M.1). 2M2, malate 2 mM supporting the anaplerotic N‐pathway F(N)*_P_*. 3P, FN{PM}*_P_*. 4G, FN*_P_*. 5S, FNS*_P_*. 6Gp, FNSGp*_P_*. 7U4, uncoupler titrations to optimum CCCP concentration 4 µM; FNSGp*_E_*. 8Rot, SGp*_E_*. 9Ama, *rox*. 10AsTm. 11Azd, *chb*. CIV activity = *J*(10AsTm) − *J*(11Azd) at closely matched O_2_ concentrations. See Tables 1, 2, 3, 4 for abbreviations. Data repository (Timón‐Gómez et al., 2024): 2021‐11‐16 P6‐02 and 2021‐11‐17 P7‐02.

Respiration of mitochondrial preparations was assessed by HRR in the Oroboros (Oroboros Instruments). The terminology used for the mitochondrial pathways is based on the type of substrate used in the experiment instead of the respiratory complex catalysing the entry of electrons into the Q‐junction (Figures 1 and 2, Table 1; Gnaiger, 2020). The 2‐mL Oroboros chambers were stirred continuously at 750 rpm containing the respiration medium MiR05, MiR06 or MiR06Cr at 37°C. Quality control criteria were applied in HRR experiments and data analysis (Baglivo et al., 2022).

**TABLE 1:** Terms and abbreviations in Figures 1 and 2.

Term	Abbreviation	Definition
Adenosine diphosphate	ADP, D	Substrate of adenine nucleotide translocase and ATP synthase of the phosphorylation system
Antimycin A	Ama	Inhibitor of Complex III
Ascorbate	As	Electron donor to TMPD in the Complex IV activity assay
Adenosine triphosphate	ATP, T	Major carrier of chemical energy in cells
Sodium azide	Azd	Inhibitor of Complex IV
Cytochrome *c*	c	Small haem protein loosely associated with the mitochondrial inner membrane transferring electrons from Complex III to Complex IV
Carbonyl cyanide *m*‐chlorophenyl hydrazone	CCCP	Uncoupler of OXPHOS
Complex I–IV	CI–CIV	Membrane‐bound enzyme complexes of the ET system
Complex of the ETF dehydrogenase	CETFDH	Membrane‐bound enzyme complex transferring electrons into the Q‐junction from ETF, linking β‐oxidation to electron entry in the Q‐junction (independent of Complex II)
Complex of glycerophosphate dehydrogenase	CGpDH	Mt‐membrane‐bound enzyme complex, oxidizing glycerophosphate to dihydroxyacetone phosphate, feeding electrons into the Q‐junction
Digitonin	Dig	Mild detergent that selectively permeabilizes the plasma membrane
Electron‐transferring flavoprotein	ETF	Small redox protein transferring reducing equivalents to CETFDH
ETS	ETS	ETS catalysing ET from reduced fuel substrates to the final acceptor O_2_, involving membrane‐bound respiratory complexes, soluble dehydrogenases and carriers.
Fatty acid pathway, FAO	F(‐pathway), FAO	Fatty acids supply electrons through fatty acyl‐CoA dehydrogenases to ETF
Glutamate	G	Amino acid that reacts with oxaloacetate to form 2‐oxoglutarate (TCA cycle) and aspartate; NADH‐linked substrate
Glycerophosphate (‐pathway)	Gp(‐pathway)	Organophosphate component of glycerophospholipids. Substrate of CGpDH to form dihydroxyacetone phosphate and feed electrons into the Q‐junction (Gp‐pathway)
Malate	M	NADH‐linked substrate, formed from fumarate in the TCA cycle
Mitochondrial respiration medium	MiR05, MiR06	Media developed for oxygraphy incubations of mitochondrial preparations; MiR06 is MiR05 supplemented with catalase
NADH(‐pathway)	N(‐pathway)	Combinations of NADH‐linked substrates (P, G, M) feed electrons into the N‐junction; NADH‐linked respiration (N‐pathway, CI‐linked) with minor contributions of the S‐pathway
Nicotinamide adenine dinucleoide	NAD	Coenzyme NAD; NADH+H^+^ plus NAD^+^
Octanoylcarnitine	Oct	Medium‐chain fatty acid covalently linked to carnitine; substrate of FAO
Pyruvate	P	Monocarboxylic acid formed in glycolysis; NADH‐linked substrate
Coenzyme Q	Q	ETS‐reactive coenzyme Q; UQ + USQ^•−^ + UQH_2_
Rotenone	Rot	Inhibitor of Complex I
Succinate(‐pathway)	S(‐pathway)	Succinate, substrate of Complex II formed in the TCA cycle; succinate‐linked respiration (S‐pathway, CII‐linked)
Tricarboxylic acid cycle	TCA	System of enzymes arranged in a cyclic metabolic arrangement in the mitochondrial matrix
*N,N,N',N'*‐tetramethyl‐*p*‐phenylenediamine dihydrochloride	TMPD, Tm	Artificial substrate that reduces cytochrome *c* in the Complex IV activity assay, reduced by ascorbate
Uncoupler	U	Protonophore which cycles across the mtIM transporting H^+^ and dissipating the protonmotive force

*Note*: The terminology adheres to the MitoEAGLE consortium paper (Gnaiger et al., 2020), and Gnaiger (2020, 2024) and Komlódi et al. (2021). Abbreviations: ETF, electron‐transferring flavoprotein; ETS, electron transfer system; FAO, fatty acid oxidation.

RP1 and RP2 were used in parallel in mitochondrial preparations (Table 2) to assess 20 respiratory states (Figure 2; Tables 3 and 4). O_2_ concentration was calibrated, and the slope computed using DatLab 7.4 or 8.1 (Oroboros Instruments). Marks were set after each titration on the oxygen slope at steady‐state to calculate respiratory rates. Background‐corrected oxygen fluxes were baseline‐corrected for *rox* (after inhibition of the ETS by antimycin A), and normalized per tissue mass (brain, liver and heart homogenate), mitochondrial protein mass (isolated cardiac mitochondria) or per cell count (HEK 293T, PBMCs and platelets).

**TABLE 2:** Living cells and mitochondrial preparations (Figure 2).

Term	Abbr.	Description
Living cells	ce	Cell population consisting of living cells (intact plasma membrane) and a small fraction of dead cells
Living to permeabilized cells	ce‐pce	Cells permeabilized in the Oroboros chamber with the mild detergent digitonin, to allow for the exchange of soluble molecules between the cytosol and respiration medium
Isolated mitochondria	imt	Separation of the mitochondria by breaking the plasma membrane of tissues or cells, followed by centrifugation steps to remove other cellular fractions
Permeabilized cells	pce	Selective permeabilization of the plasma membrane with the mild detergent digitonin without affecting the mitochondrial membranes
Tissue homogenate	thom	Mechanical permeabilization of the plasma membrane of tissue by micro‐disruption

*Note*: The terminology adheres to the MitoEAGLE consortium paper (Gnaiger et al., 2020).

**TABLE 3:** Respiratory rates in coupling control states (Figure 2).

Rate	Definition
*R*	Routine respiration of living cells regulated by physiological cellular energy demand in the range of *L* to *P*
*L*	Leak respiration, non‐phosphorylating resting rate, in the absence of ADP or presence of inhibitors of the phosphorylation system
*P*	OXPHOS capacity, maximum respiratory capacity of OXPHOS in a defined pathway state at saturating concentrations of ADP, inorganic phosphate, oxygen, and substrates; ${J_{O_{2}}}_{\max}$ in ‘cell ergometry’
*E*	ET capacity, maximum decoupled flux in a defined pathway control state at optimum concentration of a protonophore
*rox*	Residual oxygen consumption in the rox state, in the presence of inhibitors of the ETS
*ren*	Residual endogenous oxygen consumption in the ren state of mitochondrial preparations in the absence of external fuel substrates with or without ADP
*chb*	Chemical background oxygen consumption in the chb state due to autooxidation of chemicals

*Note*: *R*, *L*, *P*, and *E* are baseline‐corrected for *rox*, in contrast to total rates *R′, L′, P′*, and *E′*. *rox, ren* and *chb* are not coupling control states, but are used for baseline corrections of mitochondrial rates.

**TABLE 4:** Pathway and coupling control states or rates assessed in RP1 and RP2 in mitochondrial preparations.

Abbreviation	RP no.	Description
N{PM}*_L_*	RP1	N‐pathway with pyruvate and malate {PM} in leak state
N{PM}*_P_*	RP1	N‐pathway with pyruvate and malate in OXPHOS state
N{PM}*_E_*	RP1	N‐pathway with pyruvate and malate in ET state
N*_E_*	RP1	N‐pathway with pyruvate and glutamate and malate in ET state
NS*_E_*	RP1	Convergent N‐ and S‐pathway in ET state
FNS*_E_*	RP1	Convergent F‐, N‐ and S‐pathway in ET state
S*_E_*	RP1	S‐pathway in the presence of rotenone in ET state
F*_P_*	RP2	F‐pathway in OXPHOS state; calculation in Figure 10
F(N)*_P_*	RP2	F‐pathway in OXPHOS state in presence of 2 mM malate which may activate the N‐pathway, depending on anaplerotic capacity (e.g. mt‐malic enzyme)
FN{PM}*_P_*	RP2	Convergent F‐ and N‐pathway (with pyruvate and malate) in OXPHOS state
FN*_P_*	RP2	Convergent F‐ and N‐pathway (with pyruvate and glutamate and malate) in OXPHOS state
FNS*_P_*	RP2	Convergent F‐, N‐ and S‐pathway in OXPHOS state
FNSGp*_P_*	RP2	Convergent F‐, N‐, S‐ and Gp‐pathway in OXPHOS state
FNSGp*_E_*	RP2	Convergent F‐, N‐, S‐ and Gp‐pathway in ET state
SGp*_E_*	RP1/2	Convergent S‐ and Gp‐pathway in the presence of rotenone in ET state; harmonized step in RP1 and RP2
CIV*_E_*	RP1/2	CIV activity in ET state; harmonized step in RP1 and RP2

*Note*: Terminology from Gnaiger (2020) with some modifications of abbreviations; N{PM} or FN{PM} specifies the N‐linked substrates {PM} or {PGM}.

#### 2.4.1 SUIT reference protocols: bioenergetics design

**FIGURE 3:**
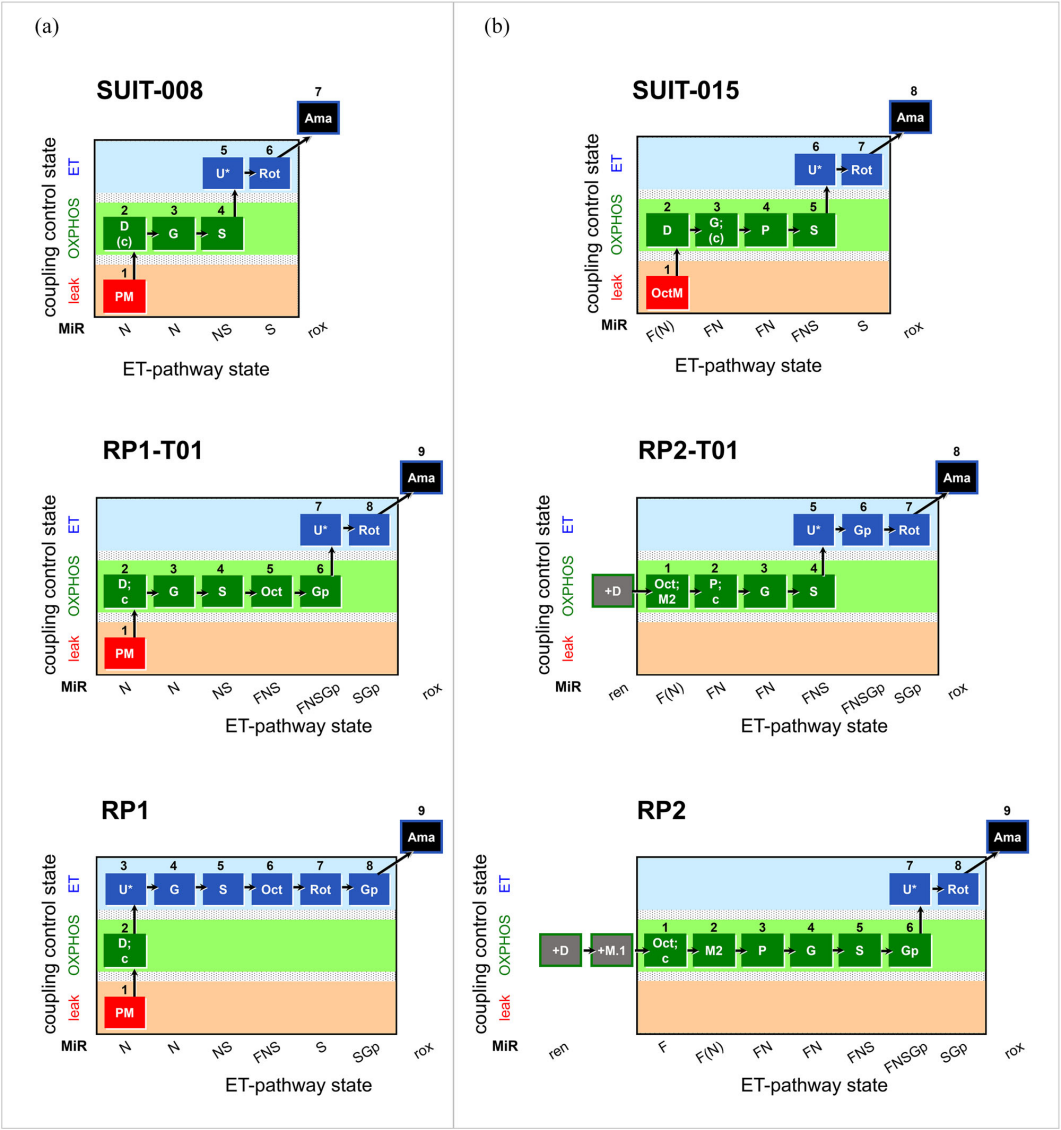
Design of the reference protocols as variations of previous SUIT protocols. Coupling control and ET pathway diagrams for the evolution of (a) RP1 and (b) RP2. (a) SUIT‐008 (Doerrier et al., 2024; Lemieux et al., 2017; Votion et al., 2012) is extended by several pathway‐control states in RP1‐T01. Both protocols address pathway control in the OXPHOS state and the excess ET capacity at maximum electron push (evaluated within a protocol) from pathways converging at the Q‐junction. These aims compromise the calculation of additivity of N‐ and S‐pathways in either the OXPHOS or ET state (Gnaiger, 2020). Therefore, RP1 was designed to analyse coupling control (leak, OXPHOS, ET) in the N‐pathway using pyruvate and malate (N{PM}*_L_*, N{PM}*_P_*_,_ N{PM}*_E_*), followed by pathway control in the ET state. (b) Different from the previously published SUIT‐015 protocol including FAO (Pesta & Gnaiger, 2012), ADP was added first in RP2‐T01. In both protocols the *E ‐ P* control efficiency was quantified in the FNS‐pathway state. In addition, titration of glycerophosphate before rotenone in RP2‐T01 allows for evaluation of a possible increase of ET capacity by stimulating the Gp‐pathway and, thus, combining even more pathways converging at the Q‐junction. When compared to RP1 with a focus on ET capacities, pathway control is physiologically more relevant in the OXPHOS state (RP2). See Section 3.3.1 for details and Tables 1, 3, and 4 for abbreviations.

RP1 and RP2 are two SUIT reference protocols for HRR. Variations of frequently applied protocols provided the rationale for the design of RP1 and RP2 (Figure 3). RP1 begins with a sequence of coupling control steps in the N‐pathway. PM was selected because the contribution of the S‐pathway with N‐linked substrates is lower with PM than with GM (Sumbalová et al., 2023). A defect of the N‐pathway may be localized when glutamate boosts the N‐pathway and compensates for damage in the pyruvate pathway upstream of CI. The addition of succinate addresses the physiological core of partially additive electron flow at the Q‐junction (Gnaiger, 2020).

In RP1, octanoylcarnitine is added after the combined substrates for the NS‐pathway to evaluate a possible contribution of FAO to maximal respiratory capacity (e.g., during exercise). Rotenone inhibits not only the N‐ but also the F‐pathway, and thus, RP1 addresses the N+S additivity in the ET state, comparing the arithmetic sum of the single pathway fluxes (N*_E_* + SRot*_E_*) with the combined pathway flux NS*_E_* (Gnaiger, 2020). Glycerophosphate addition (SGp*_E_* in Figure 2a) does not provide information on the single Gp‐pathway nor about a stimulatory effect above NS‐pathway capacity, but SGp*_E_* yields a comparable (harmonized) state in RP1 and RP2. Finally, *rox* is measured after the addition of antimycin A in both RP1 and RP2.

**FIGURE 4:**
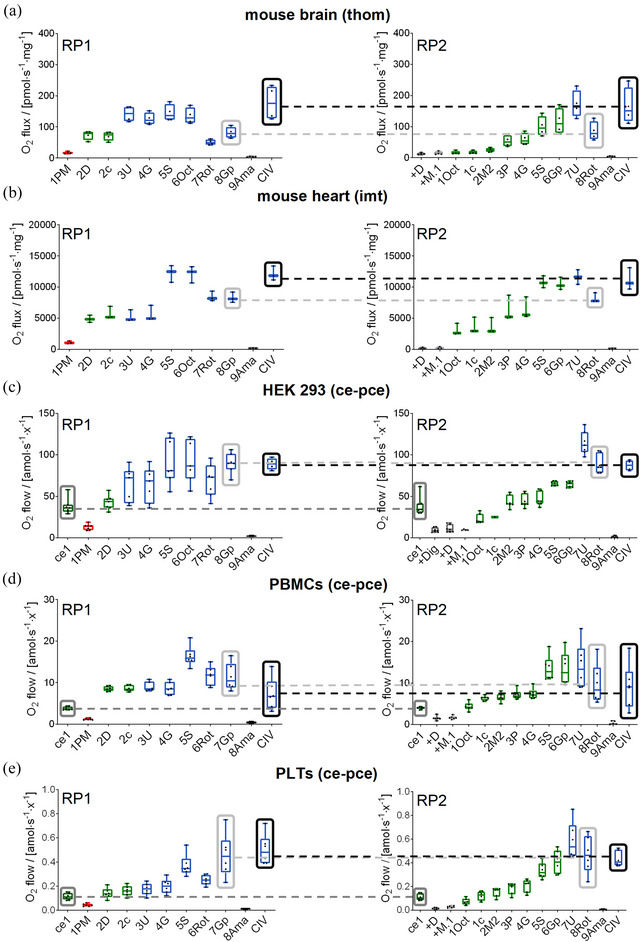
RP1 and RP2 for precision OXPHOS analysis of mitochondrial preparations from mouse and human models. O_2_ fluxes (pmol s^−1^ mg^−1^) or flows (amol s^−1^ x^−1^), calculated as O_2_ flux per *V* (pmol s^−1^ mL^−1^) divided by tissue wet mass concentration (a), protein mass concentration (b), or cell count concentration (c–e); obtained in RP1 and RP2. Results are expressed as median with the interquartile range. Comparable states in RP1 and RP2 are shown by grey squares connected by dashed grey lines. Representative traces are shown in Figure 2 (HEK 293T), Figure 5 (mouse heart and brain), and Figure 6 (PBMCs and platelets). (a) Brain tissue homogenate (thom) from mouse, *N *= 4. (b) Cardiac isolated mitochondria (imt) from mouse, *N *= 3. (c) Permeabilized HEK 293T cells (pce), *N *= 7. (d) Human PBMCs, *N *= 6. (e) Human platelets, *N *= 6. Data repository (Timón‐Gómez et al., 2024): (a) RP1: 2017‐02‐06 P6‐02, 2017‐02‐20 P4‐02, 2017‐02‐21 P5‐02, 2017‐03‐28 P5‐02; RP2: 2017‐02‐06 P6‐02, 2017‐02‐20 P3‐02, 2017‐02‐21 P6‐02, 2017‐03‐28 P6‐02. (b) RP1: 2017‐02‐06 P1‐02, 2017‐02‐08 P2‐02, 2017‐02‐08 P3‐02; RP2: 2017‐02‐06 P2‐02, 2017‐02‐08 P1‐02, 2017‐02‐08 P4‐02. (c) RP1&RP2: 2017‐02‐23 P8‐03, 2017‐05‐09 P3‐02, 2017‐12‐05 P1‐03, 2017‐06‐28 P1‐05, 2017‐06‐28 P2‐03, 2017‐06‐28 P3‐06, 2017‐06‐28 P4‐03, 2017‐06‐28 P5‐03, 2017‐06‐28 P6‐03, 2017‐06‐28 P7‐03. (d) RP1&RP2: 2016‐09‐26 PS4‐02, 2016‐09‐28 PS3‐02, 2016‐09‐28 PS4‐01, 2016‐09‐29 PS3‐02, 2016‐09‐29 PS4‐02, 2016‐10‐05 PS4‐02. (e) RP1&RP2: 2016‐09‐26 PS2‐02, 2016‐09‐28 PS5‐02, 2016‐09‐28 PS6‐02, 2016‐09‐29 PS5‐02, 2016‐09‐29 PS6‐02, 2016‐10‐05 PS7‐02.

In RP2, FAO capacity is measured first (Section 2.4.4). NADH‐linked substrates, succinate and glycerophosphate are added sequentially in the OXPHOS state, which is physiologically more relevant than the ET state in RP1. Then, rotenone added after glycerophosphate yields the comparable SGp*_E_* state in RP2 and RP1 (Figures 2 and 4).

**FIGURE 5:**
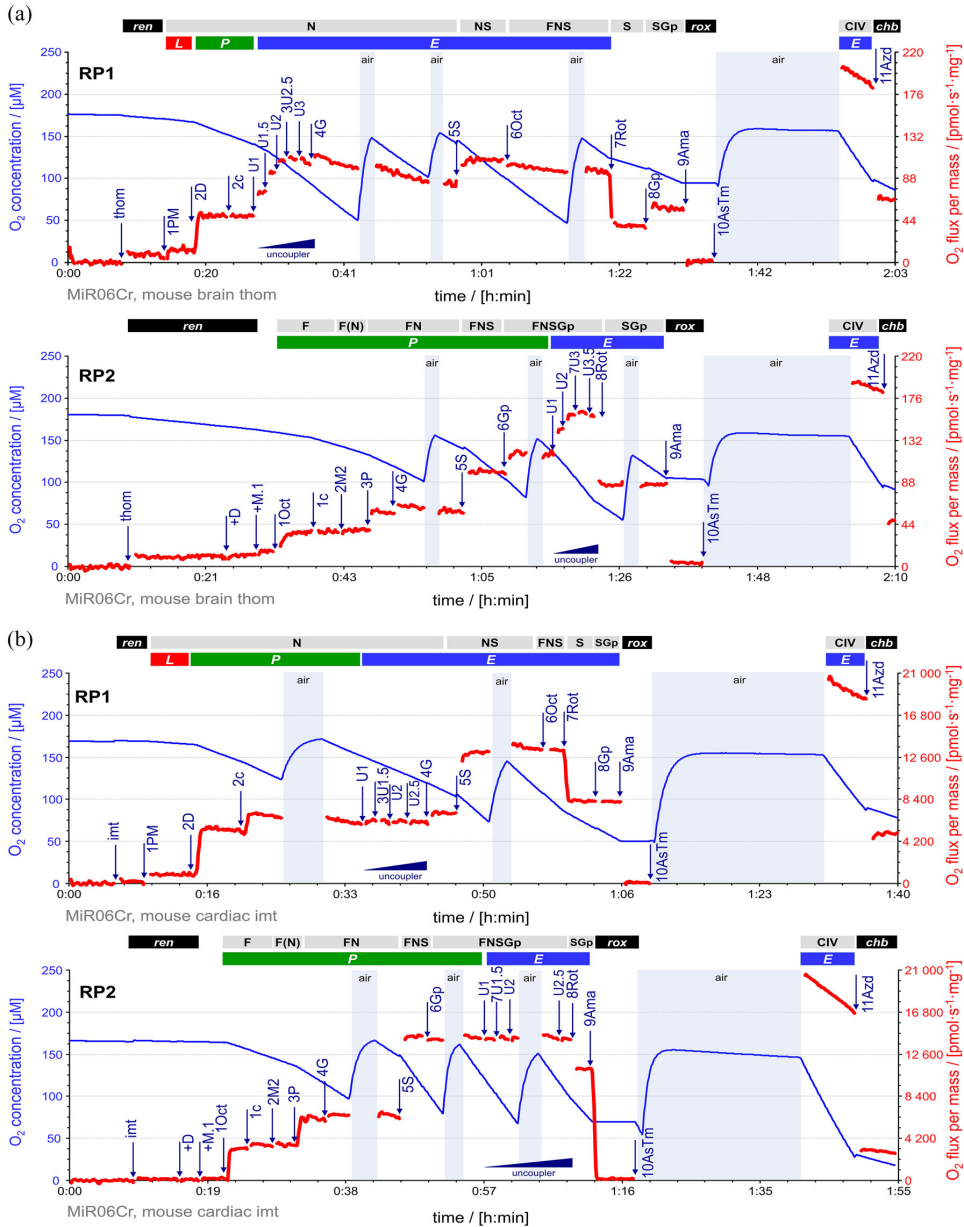
RP1 and RP2 for mouse tissues. Representative traces of O_2_ concentration (µM) (blue line) and O_2_ flux per mass (pmol s^−1^ mg^−1^) (red line), calculated as O_2_ flux per *V* (pmol s^−1^ mL^−1^) divided by (a) tissue wet mass concentration in mouse brain homogenate or by (b) mitochondrial protein concentration in mouse cardiac mitochondria. Titration spikes were eliminated. Oxygen concentration was kept above 50 µM by intermittent reoxygenations (air, open chamber, blue shade). (a) Sequence of respiratory states in brain in RP1, characterized by titrations and corresponding rates: mt, *ren*. 1PM, N*_L_*. 2D, N*_P_*. 2c, cytochrome *c* (cyt *c*); N[c]*_*P*_*. 3U2.5, uncoupler titrations to optimum CCCP concentration 2.5 µM; N*_E_*. 4G, N{PGM}*_E_*. 5S, NS*_E_*. 6Oct, FNS*_E_*. 7Rot, S*_E_*. 8Gp, SGp*_E_*. 9Ama, *rox*. 10AsTm, substrates for CIV stimulation; the chamber was kept open for 20 min to allow for redox equilibration without depletion of oxygen (Doerrier et al., 2018); CIV*_E_*. 11Azd, *chb*. Sequence of respiratory states in RP2: mt, *ren*. +D, stimulating *ren*. +M.1, 1Oct and 1c, F*_P_* = *J*(1Oct[c]) − *J*(+M.1). 2M2, F(N)*_P_*. 3P and 4G, FN*_P_*. 5S, FNS*_P_*. 6Gp, FNSGp*_P_*. 7U3, uncoupler titrations to optimum CCCP concentration 3 µM; FNSGp*_E_*. 8Rot, SGp*_E_*. 9Ama, *rox*. 10AsTm, CIV*_E_*. 11Azd, *chb*. (b) Sequence of respiratory states in heart in RP1, characterized by titrations and corresponding rates: mt, *ren*. 1PM, N*_L_*. 2D, N*_P_*. 2c, N[c]*_*P*_*. 3U1.5, uncoupler titrations to optimum CCCP concentration 1.5 µM; N*_E_*. 4G, N{PGM}*_E_*. 5S, NS*_E_*. 6Oct, FNS*_E_*. 7Rot, S*_E_*. 8Gp, SGp*_E_*. 9Ama, *rox*. 10AsTm, CIV*_E_*. 11Azd, *chb*. Sequence of respiratory states in RP2: mt, *ren*. +D, stimulating *ren*. +M.1, 1Oct and 1c, F*_P_* = *J*(1Oct[c]) − *J*(+M.1). 2M2, F(N)*_P_*. 3P and 4G, FN*_P_*. 5S, FNS*_P_*. 6Gp, FNSGp*_P_*. 7U1.5, uncoupler titrations to optimum CCCP concentration 1.5 µM; FNSGp*_E_*. 8Rot, SGp*_E_*. 9Ama, *rox*. 10AsTm, CIV*_E_*. 11Azd, *chb*. CIV activity = *J*(10AsTm) − *J*(11Azd) at closely matched O_2_ concentrations. See Figure 2 for details and Table 4 for abbreviations. Data repository (Timón‐Gómez et al., 2024): 2017‐02‐06_P1‐02, 2017‐02‐08_P1‐02, 2017‐02‐08_P11‐02, 2017‐03‐28_P6‐02.

**FIGURE 6:**
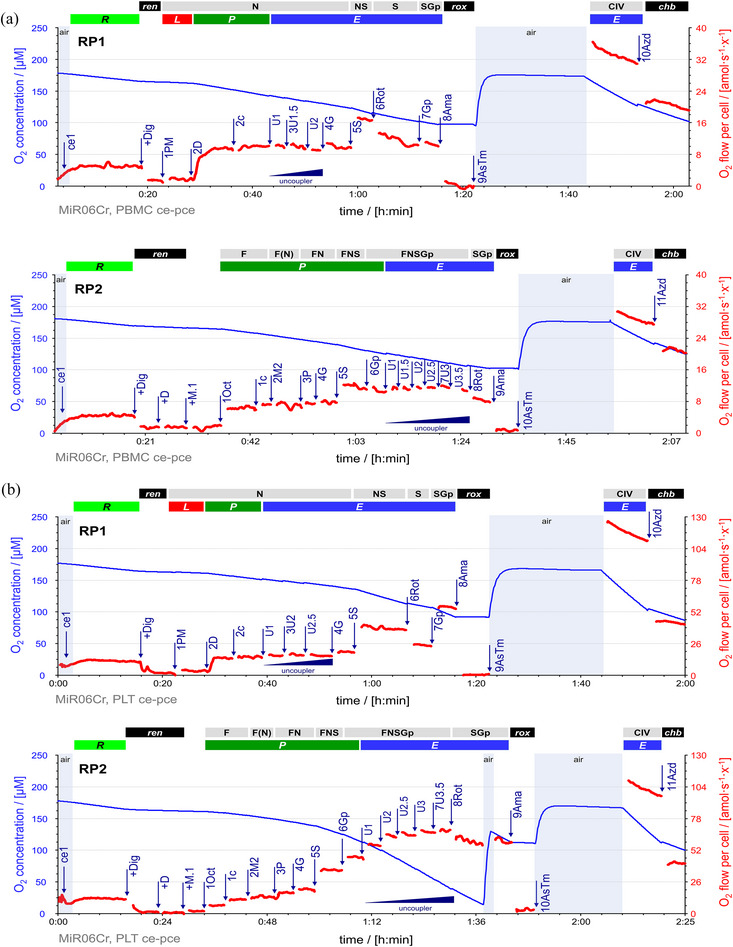
RP1 and RP2 for blood cells. Representative traces of O_2_ concentration (µM) (blue line) and O_2_ flow (amol s^−1^ x^−1^) (red line), calculated as O_2_ flux per *V* (pmol s^−1^ mL^−1^) divided by cell count concentration. Titration spikes were eliminated. Oxygen concentration was kept above 100 µM (except one case at 30 µM) by intermittent reoxygenations (air, open chamber, blue shade). (a) Sequence of respiratory states in RP1, characterized by titrations and corresponding rates in PBMCs: ce1, *R*. +Dig, digitonin 16 µg mL^−1^; *ren*. 1PM, N*_L_*. 2D, 1 mM ADP; N*_P_*. 2c, N[c]*_*P*_*. 3U1.5, uncoupler titrations to optimum CCCP concentration 1.5 µM; N*_E_*. 4G, N{PGM}*_E_*. 5S, NS*_E_*. 6Rot, S*_E_*. 7Gp, SGp*_E_*. 8Ama, *rox*. 9AsTm, CIV*_E_*. 10Azd, *chb*. Sequence of respiratory states in RP2: ce1, *R*. +Dig, digitonin 16 µg mL^−1^; *ren*. +D, 1 mM ADP, stimulating *ren*. +M.1, 1Oct and 1c, F*_P_* = *J*(1Oct[c]) − *J*(+M.1). 2M2, F(N)*_P_*. 3P and 4G, FN*_P_*. 5S, FNS*_P_*. 6Gp, FNSGp*_P_*. 7U3, uncoupler titrations to optimum CCCP concentration 3 µM; FNSGp*_E_*. 8Rot, SGp*_E_*. 9Ama, *rox*. 10AsTm, CIV*_E_*. 11Azd, *chb*. (b) Sequence of respiratory states in RP1, characterized by titrations and corresponding rates in platelets: ce1, *R*. +Dig, digitonin 16 µg mL^−1^; *ren*. 1PM, N*_L_*. 2D, 1 mM ADP; N*_P_*. 2c, N[c]*_*P*_*. 3U2, uncoupler titrations to optimum CCCP concentration 2 µM; N*_E_*. 4G, N{PGM}*_E_*. 5S, NS*_E_*. 6Rot, S*_E_*. 7Gp, SGp*_E_*. 8Ama, *rox*. 9AsTm, CIV*_E_*. 10Azd, *chb*. Sequence of respiratory states in RP2: ce1, *R*. +Dig, digitonin 16 µg mL^−1^; *ren*. +D, 1 mM ADP, stimulating *ren*. +M.1, 1Oct and 1c, F*_P_* = *J*(1Oct[c]) − *J*(+M.1). 2M2, F(N)*_P_*. 3P and 4G, FN*_P_*. 5S, FNS*_P_*. 6Gp, FNSGp*_P_*. 7U3.5, uncoupler titrations to optimum CCCP concentration 3.5 µM; FNSGp*_E_*. 8Rot, SGp*_E_*. 9Ama, *rox*. 10AsTm, CIV*_E_*. 11Azd, *chb*. CIV activity = *J*(AsTm) − *J*(Azd) at closely matched O_2_ concentrations. See Figure 2 for description of the sequence of respiratory states and Table 4 for abbreviations. Data repository (Timón‐Gómez et al., 2024): 2016‐09‐28_PS4_01 and 2016‐09‐29_PS5_02.

**FIGURE 7:**
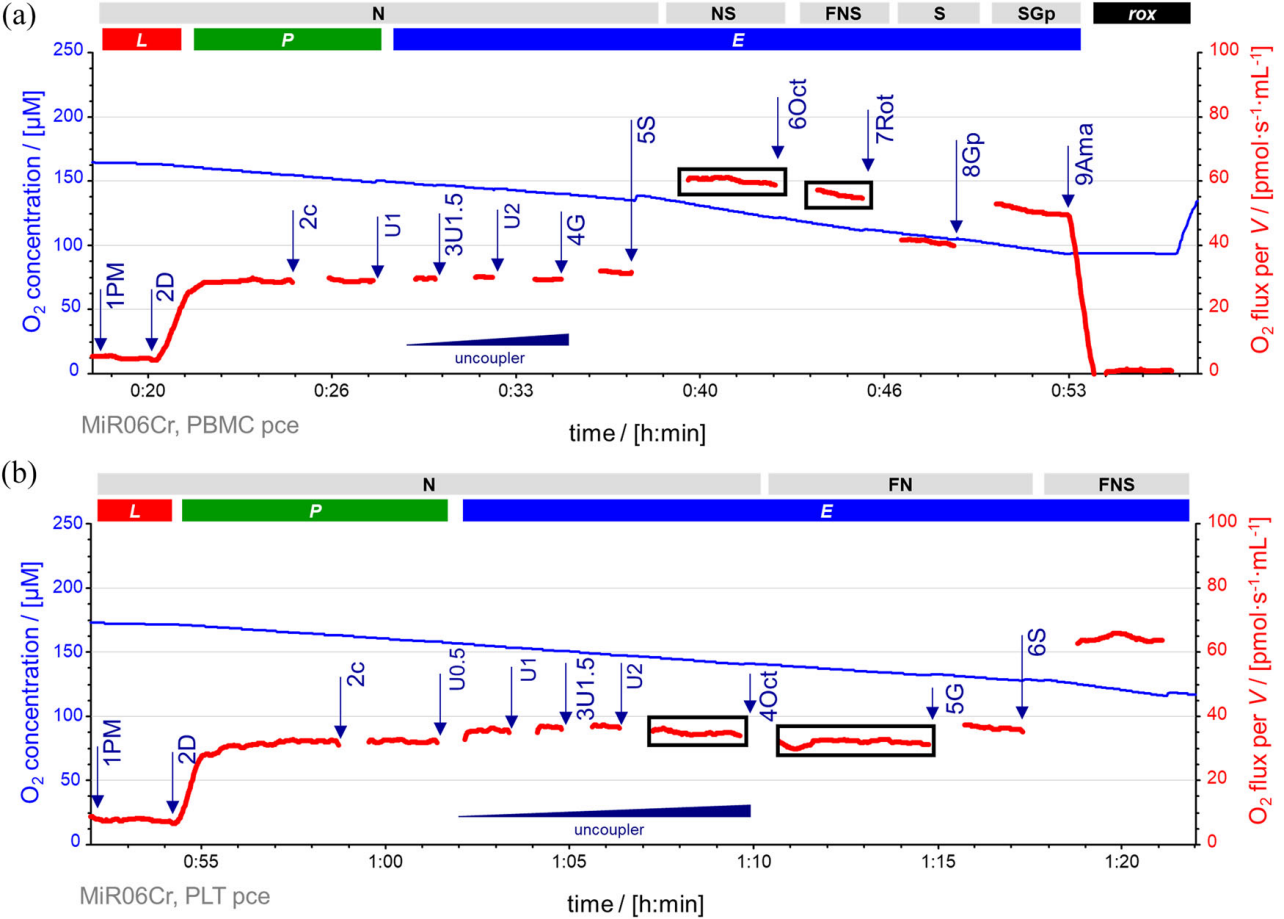
Inhibitory effect of octanoylcarnitine in blood cells in the N‐ and NS‐pathway. Representative traces of O_2_ concentration (µM) (blue line) and O_2_ flux per *V* (pmol s^­1^ mL^−1^) (red line). Titration spikes were eliminated. (a) Section of RP1 to assess the effect of Oct in the NS‐pathway in PBMCs. See Figure 2 for the complete sequence and concentration of chemicals. 1PM, N*_L_*. 2D, 1 mM ADP; N*_P_*. 2c, N[c]_*P*_. 3U1.5, uncoupler titrations to optimum CCCP concentration at 1.5 µM; N*_E_*. 4G; N*_E_*. 5S, NS*_E_*. 6Oct, FNS*_E_*. 7Rot, S*_E_*. 8Gp, SGp*_E_*. 9Ama, *rox*. (b) Variation of RP1 to test the effect of Oct in the N‐pathway in platelets. Sequence of rates (titrations and states): 1PM, N*_L_*. 2D, 1 mM ADP; N*_P_*. 2c, N[c]_P_. 3U1.5, uncoupler titrations to optimum CCCP concentration at 1.5 µM; N*_E_*. 4Oct; FN{PM}*_E_*. 5G; FN{PGM}*_E_*. 6S, FNS*_E_*. See Table 4 for abbreviations. Data repository (Timón‐Gómez et al., 2024): 2016‐01‐29 P4‐02, 2016‐05‐25 P7‐03a.

The *E ‐ P* control efficiency is the normalized *E ‐ P* excess capacity calculated as (*E − P*)/*E*. At zero *E ‐ P* control efficiency, the capacity of the phosphorylation system does not exert any limitation on *P*. When multiple Q‐junction pathways exert an additive effect on *E*, the capacity of the phosphorylation system may be exceeded, which is detected as increased *E ‐ P* control efficiency (Cardoso & Gnaiger, 2024). In RP1, the *E ‐ P* control efficiency in the N‐pathway might underestimate the control of OXPHOS capacity by the phosphorylation system in the NS‐ or FNS‐pathway state. In contrast, in RP2 the transition from OXPHOS‐ to ET‐capacity takes place at maximum electron push from multiple pathways (FNSGp). Representative traces are shown for permeabilized HEK 293T cells (Figure 2b), mouse brain homogenate (Figure 5a) and cardiac isolated mitochondria (Figure 5b), and permeabilized PBMCs (Figures 6a and 7a) and platelets (Figure 6b and 7b).

**FIGURE 8:**
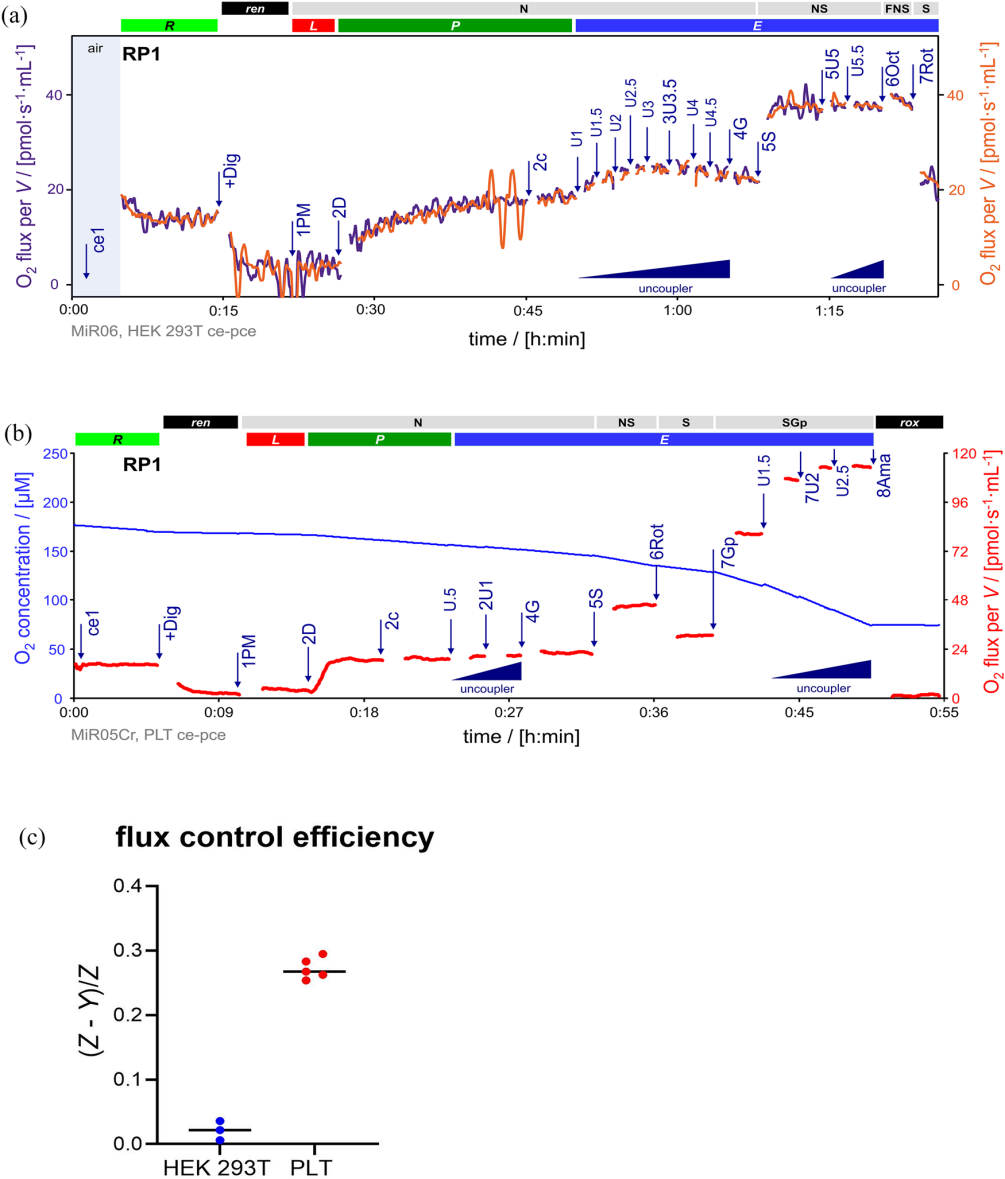
A second uncoupler titration after substrate combinations might be needed to obtain ET capacity in some tissues/cell types. The second addition of uncoupler was performed after the substrate combination that had higher oxygen fluxes for each cell type. (a) Representative traces of the overlay of O_2_ fluxes per *V* (pmol s^−1^ mL^−1^) (purple and orange lines) from two technical repeats of a variation of RP1 in HEK 293T cells. Titration spikes were eliminated. Sequence of respiratory states, characterized by titrations and corresponding rates: ce1, *R*. +Dig, *ren*. 1PM, N*_L_*. 2D, N*_P_*. 2c, N[c]_*P*_. 3U3.5, uncoupler titrations to optimum CCCP concentration 3.5 µM; N*_E_*. 4G, N{PGM}*_E_*. 5S, NS*_E_*. 5U.5, second uncoupler titrations to optimum CCCP concentration 0.5 µM, NS*_E_*, not increasing oxygen fluxes. 6Oct, FNS*_E_*. 7Rot, S*_E_*. (b) Representative traces of O_2_ concentration (µM) (blue line) and O_2_ flux per *V* (pmol s^−1^ mL^−1^) of a variation of RP1 in platelets. Titration spikes were eliminated. Sequence of respiratory states, characterized by titrations and corresponding rates: ce1, *R*. +Dig, *ren*. 1PM, N*_L_*. 2D, N*_P_*. 2c, N[c]_*P*_. 3U1, uncoupler titrations to optimum CCCP concentration 1 µM; N*_E_*. 4G, N{PGM}*_E_*. 5S, NS*_E_*. 6Rot, S*_E_*. 7Gp, SGp*_E_*. 7U1, second set of uncoupler titrations to optimum CCCP concentration 2 µM, to reach maximum ET capacity SGp*_E_*. 8Ama, *rox*. (c) Flux control efficiencies of the second addition of uncoupler in HEK 293T (*N *= 3, *n *= 2) and platelets (*N *= 5, *n *= 1). In HEK 293T cells, *J*_1_(5U) and *J*_2_(5S). In PLTs, *J*_1_(7U) and *J*_2_(7Gp). See Table 4 for abbreviations. Data repository (Timón‐Gómez et al., 2024): (a) 2024‐03‐19_Q‐0009_03, 2024‐03‐20_Q‐0009_03, and 2024‐03‐20_Q‐0005_03. (b) 2024‐06‐10_P1‐02, 2024‐06‐12_P1‐02, 2024‐06‐13_P1‐02, 2024‐06‐13_P1‐04, 2024‐06‐13_P2‐03.

The uncoupler concentration titrated to reach ET capacity in the N‐pathway; however, might not be optimal in subsequent states of substrate combinations. An additional uncoupler titration may then be necessary to reach maximum ET capacity, as suggested when the respiratory rates in the harmonized states SGp*_E_* differ between RP1 and RP2. Additional uncoupler titrations are not always necessary (Figure 8).

#### 2.4.2 Leak respiration

In RP1 and RP2, oligomycin is not used to induce the leak state, as compared to other protocols (Gnaiger et al., 2015; Hutter et al., 2004; Krumschnabel et al., 2014). Oligomycin particularly at high concentration (in the µM range) exerts an inhibitory effect on ET capacity in several cell types (Doerrier et al., 2018; Ruas et al., 2016, 2018). In contrast, the leak rate is assessed in RP1 after addition of N‐linked substrates without ADP. Oxygen flux in the leak state in the absence of external adenylates is identical to flux after inhibition of ATP synthase by oligomycin (Krumschnabel et al., 2014). Measurements of O_2_ flux in the consecutive leak, OXPHOS and ET states are performed at constant N‐linked pathway control in RP1, to avoid any potential confounding substrate competition effects on coupling control.

#### 2.4.3 Complex IV activity

Both RP1 and RP2 can include measurement of cytochrome *c* oxidase (CIV) activity after inhibition of CIII by antimycin A, in the presence of 10 µM cytochrome *c* (Figure 2). Ascorbate (reduces TMPD) and TMPD (a cytochrome *c* electron donor) reduce cytochrome *c*, which, in turn, transfers electrons to CIV at a constant rate. This assay is performed in the ET state, to avoid any potential control by the phosphorylation system. The total oxygen consumption observed under these conditions is not only due to CIV activity but includes the oxygen‐dependent autoxidation of ascorbate and TMPD. The concentrations of TMPD and cytochrome *c* in this assay are below kinetic saturation to avoid excessively high autoxidation (Gnaiger et al., 1998; Gnaiger & Kuznetsov, 2002).

**FIGURE 9:**
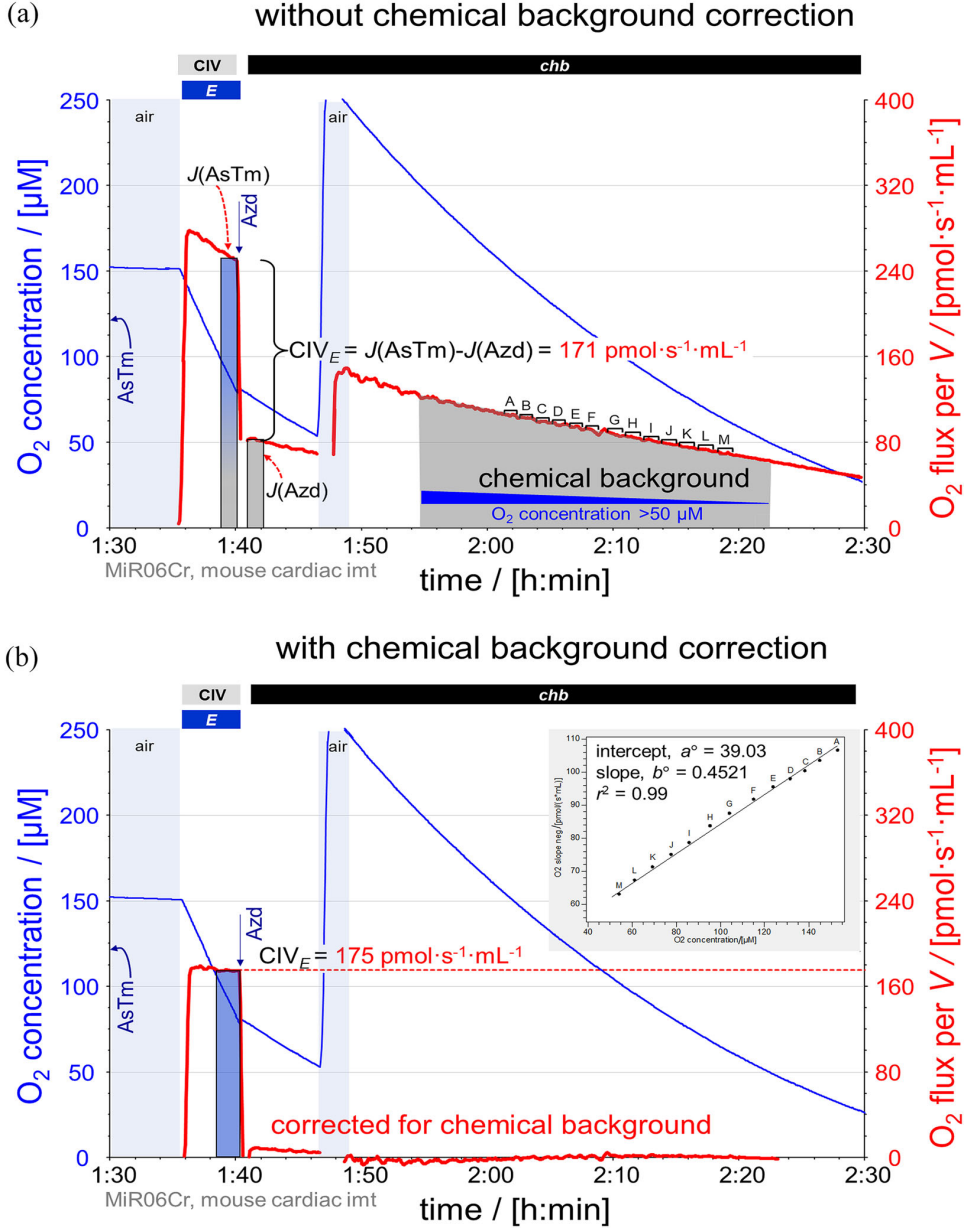
Evaluation of cytochrome *c* oxidase (CIV) activity. Representative traces of O_2_ concentration (µM) (blue line) and O_2_ flux per *V* (pmol s^−1^ mL^−1^) (red line), in mouse cardiac mitochondria. Titration spikes were eliminated. CIV activity measured in the presence of cytochrome *c*, antimycin A, ascorbate and TMPD (see Figure 2 for concentrations of chemicals). AsTm, substrates for CIV stimulation; the chamber was kept open for 20 min to allow for redox equilibration without depletion of oxygen (Doerrier et al., 2018). Azd, sodium azide 200 mM inhibiting CIV; oxygen‐dependent chemical background flux *chb*. The chamber was open to reoxygenate (air, blue shade) and closed to measure *chb* at different oxygen concentrations. Marks used for the chemical background correction are labelled as A‐O. (a) CIV activity calculated as CIV*_E_* = *J*(AsTm) − *J*(Azd) at closely matched O_2_ concentrations (marks in green). (b) CIV activity after chemical background correction using the software DatLab 7.4. Marks A‐O were selected in the ‘Background correction’ window, obtaining the graph shown on the upper right corner, and used to calibrate for the chemical background. See Table 4 for abbreviations. Data repository (Timón‐Gómez et al., 2024): 2016‐01‐26_P10‐02_1, 2016‐01‐26_P10‐02_2.

After the addition of ascorbate and TMPD, the Oroboros chamber was kept open for 20 min for redox equilibration of the chemicals without depletion of oxygen (Figure 2b). Sodium azide was added 5 min after closing the chamber. Inhibitors of CIV (e.g., sodium azide, cyanide) are used to evaluate the chemical O_2_ background flux due to autoxidation of ascorbate and TMPD. Sodium azide is used in RP1 and RP2 because inhibition of CIV by cyanide is competitively reduced by pyruvate at high oxygen levels. Autoxidation is a linear function of oxygen concentration above 50 µM O_2_ (https://wiki.oroboros.at/index.php/Chemical_O2_background; accessed 4 March 2025). Therefore, oxygen flux after inhibition of CIV is subtracted from the flux stimulated by ascorbate and TMPD at similar O_2_ concentrations. As a control, DatLab provides both an instrumental and chemical background correction when O_2_ concentrations are kept above 50 µM in the measurement of CIV activity (Figure 9).

#### 2.4.4 Fatty acid oxidation capacity

**FIGURE 10:**
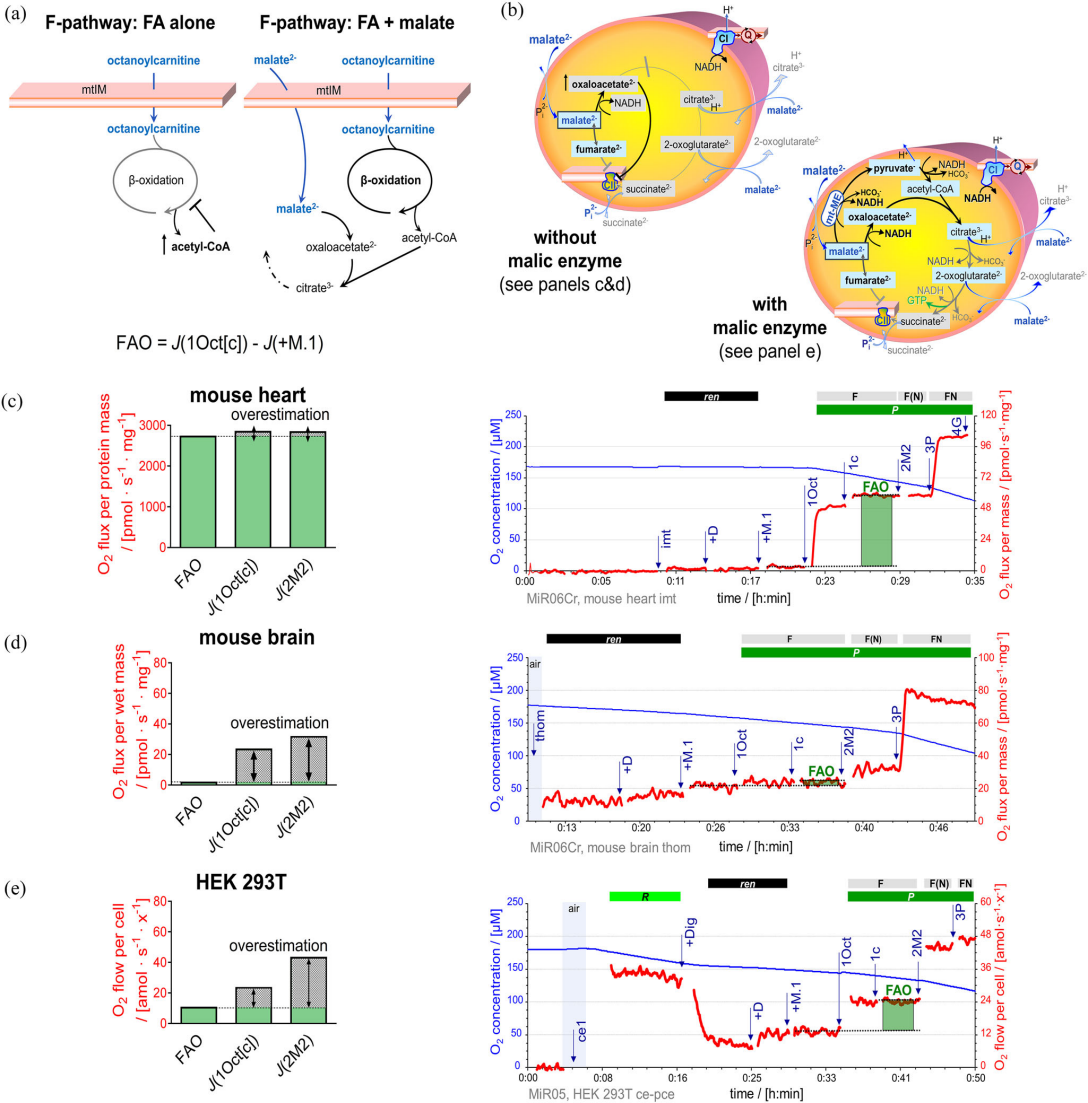
Analysis of anaplerotic reactions in RP2. (a) Evaluation of FAO requires the combination of fatty acid(s) and malate to avoid the accumulation of acetyl‐CoA, which inhibits FAO. (b) Malate alone supports mitochondrial respiration in the presence of anaplerotic pathways (e.g., mitochondrial malic enzyme, mt‐ME). (c–e) RP2 design takes into consideration anaplerotic pathways that generate pyruvate from malate (Bernstine et al., 1979; Sauer et al., 1980), replenishing the TCA cycle intermediates. To measure F‐linked respiration in any mitochondrial preparation, malate is added at low concentration (0.1 mM) in combination with octanoylcarnitine. Overestimation of FAO is shown as *J*(1Oct[c]) or *J*(2M2) versus FAO evaluation determined by *J*(1Oct[c]) − *J*(+M.1) in (c) mouse cardiac isolated mitochondria, (d) mouse brain tissue homogenate, and (e) permeabilized HEK 293T cells. Representative traces of a section of RP2 are shown for each model with O_2_ concentration (µM) (blue line) and O_2_ fluxes (pmol s^−1^ mg^−1^) or flows (amol s^−1^ x^−1^) (red line), calculated as O_2_ flux per *V* (pmol s^−1^ mL^−1^) divided by concentrations of protein mass (c), tissue wet mass (d), or cell count (e). Titration spikes were eliminated. Sequence of respiratory states, characterized by titrations and corresponding rates (see Figure 2 for complete sequence in RP2): (c) imt, isolated mitochondria; *ren*. +D, stimulating *ren*. +M.1, 1Oct and 1c, F*_P_* = *J*(1Oct[c]) − *J*(+M.1). 2M2, F(N)*_P_*. 3P, FN{PM}*_P_*. 4G, FN*_P_*. (d) thom, tissue homogenate; *ren*. +D, stimulating *ren*. +M.1, 1Oct and 1c, F*_P_* = *J*(1Oct[c]) − *J*(+M.1). 2M2, F(N)*_P_*. 3P, FN{PM}*_P_*. (e) ce1, *R*. +Dig, *ren*. +D, stimulating *ren*. +M.1, 1Oct and 1c, F*_P_*. 2M2, F(N)*_P_*. 3P, FN{PM}*_P_*. See Table 4 for abbreviations. Data repository (Timón‐Gómez et al., 2024): 2017‐02‐08 P4‐02, 2017‐03‐28 P6‐02, 2021‐11‐17 P7‐02.

The RP2 protocol was designed to evaluate primarily FAO in relation to N‐ and NS‐pathway capacity in the OXPHOS state (Figure 2). ADP is added first to deplete endogenous substrates by activating their oxidation (Chance & Williams, 1955), which is more important for defining F*_P_* compared to N*_P_*. In this way, the *L* ‐ *P* coupling control step is omitted which, however, is addressed in RP1. Two millimolar malate added in combination with octanoylcarnitine or fatty acids avoids inhibition of FAO by accumulation of acetyl‐CoA (Figure 10a; Gnaiger et al., 2015; Lemmi et al., 1990; Osiki et al., 2016; Smith et al., 2012), but may lead to overestimation of F‐pathway capacity if mitochondrial malic enzyme or other anaplerotic pathways produce pyruvate from malate simultaneously with oxaloacetate (Figure 10b–e; Sauer et al., 1980). This is especially important in proliferating cells, for example, cancer cell lines (Jiang et al., 2013). To avoid activation of the malate–anaplerotic pathway, malate is added at a low concentration (0.1 mM) before octanoylcarnitine (Figure 2b).

**FIGURE 11:**
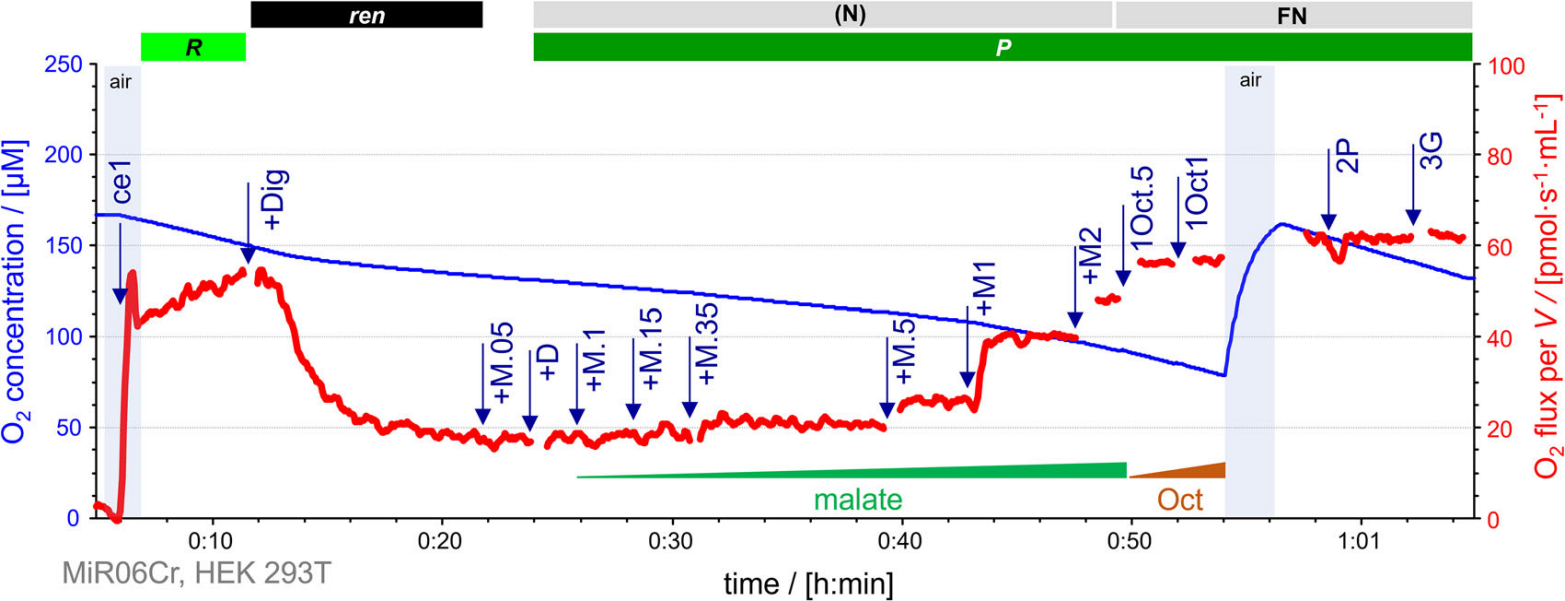
Anaplerotic pathways in HEK 293T cells. Malate alone does support mitochondrial respiration in the presence of the mtME in HEK 293T cells. Representative traces of O_2_ concentration (µM) (blue line) and O_2_ flux per *V* (pmol s^−1^ mL^−1^) (red line). Titration spikes were eliminated. Oxygen concentration was kept above 70 µM by intermittent reoxygenations (air, open chamber, blue shade). Sequence of respiratory states, characterized by titrations and corresponding rates: ce1, *R*. +Dig, *ren*. +M.05, malate 0.05 mM. +D, stimulating *ren*. +M.1, +M.15, +M.35, +M.5, +M1, +M2, increasing concentrations of malate (0.1, 0.15, 0.35, 0.5, 1 and 2 mM, respectively) to observe stepwise stimulation of respiration by mtME in HEK 293T cells; (N)*_P_*. 1Oct.5 and 1Oct1, 0.5 and 1 mM octanoylcarnitine, to test the optimal concentration of fatty acid; F(N)*_P_*. 2P and 3G, FN*_P_*. See Table 4 for abbreviations. Data repository (Timón‐Gómez et al., 2024): 2016‐01‐15 P8‐02. mtME, mitochondrial malic enzyme.

Anaplerotic pathways, endogenous substrates of mitochondrial preparations, and the quality of the mitochondrial preparation (cytochrome *c* control efficiency, see next section) may lead to over‐ or underestimation of F‐pathway capacity. This is avoided by subtracting the oxygen flux measured in the presence of low malate from the octanoylcarnitine flux after cytochrome *c* addition: *J*(1Oct[c]) – *J*(+M.1) (Figures 10 and 11; Section 2.4.5).

#### 2.4.5 Quality control of mitochondrial preparations

**FIGURE 12:**
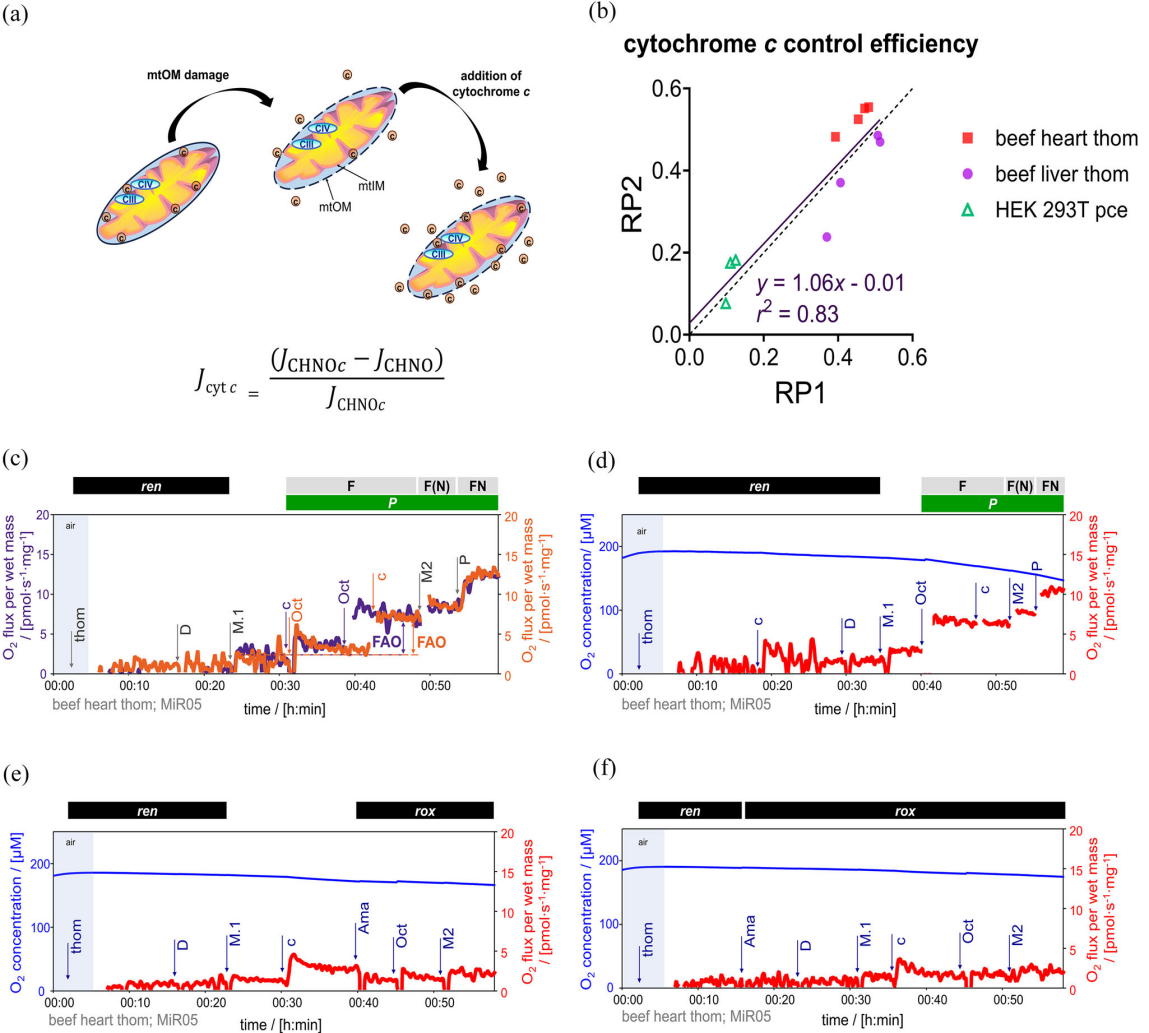
Control of the integrity of the mtOM in RP1 and RP2. (a) Damage in the mtOM causes a release of cytochrome *c*, decreasing respiration. To not underestimate respiratory capacities, a titration of saturating concentrations of cytochrome *c* is performed to detect cytochrome *c* loss from the mitochondrial intermembrane space. (b) Cluster analysis of cytochrome *c* efficiencies calculated in HEK 293T cells (*N *= 3) and beef heart and liver homogenate (*N *= 4) (models of induction of cytochrome *c* loss, see Methods section). Dots represent cytochrome *c* control efficiencies calculated from RP1 and RP2 performed in parallel. Dashed black line: theoretical line of correspondence. Solid line and equation: inverted regression analysis of the efficiencies. (c–f) Representative traces from the same beef heart homogenate of O_2_ concentration (µM) (blue line) and O_2_ fluxes (pmol s^−1^ mg^−1^) (red line), calculated as O_2_ flux per *V* (pmol s^−1^ mL^−1^) divided by tissue wet mass concentration. Titration spikes were eliminated. At least two biological replicates were assessed with each protocol (*N *= 2, *n *= 2). (c) FAO calculation is not altered by the addition of cytochrome *c* before octanoylcarnitine. Overlay of O_2_ fluxes of two SUIT protocols shown in orange and purple lines, with common titrations in grey. Sequence of respiratory states shown in orange, characterized by titrations and corresponding rates: thom, *ren*. +D, stimulating *ren*. +M.1, 1Oct and 1c, F*_P_* = *J*(1Oct[c]) − *J*(+M.1). 2M2, F(N)*_P_*. 3P, FN*_P_*. Sequence shown in purple: thom, *ren*. +D, stimulating *ren*. +M.1, +c, 1Oct, F*_P_* = *J*(1Oct) − *J*(+M.1). 2M2, F(N)*_P_*. 3P, FN*_P_*. (d–f) Cytochrome *c* respirometric artifacts. Cytochrome *c* effect on respiration is difficult to assess in these situations. (d) Sequence of respiratory states to observe cytochrome *c* effect in leak state: thom, *ren*. +c. +D, stimulating *ren*. +M.1 and 1Oct, F*_P_* = *J*(1Oct) − *J*(+M.1). 1c, second titration of cytochrome *c*, to confirm previous saturating concentration. 2M2, F(N)*_P_*. 3P, FN*_P_*. (e) Sequence of respiratory states adding cytochrome *c* after low concentration of malate: thom, *ren*. +D, stimulating *ren*. +M.1. +c. 1Ama, 1Oct and 1M2, *rox*. (f) Cytochrome *c* effect evaluated in *rox* state. Sequence of respiratory states: thom, *ren*. 1Ama, 1D, 1 M.1, 1c, 1Oct and 1M2, *rox*. See Figure 2 for concentrations of chemicals. See Table 4 for abbreviations. Data repository (Timón‐Gómez et al., 2024): 2023‐05‐16_Q3‐005_02, 2023‐05‐16_Q3‐001_02, 2023‐05‐16_Q3‐004_03, 2023‐02‐21_P5_02, 2023‐02‐21_Q‐0009_02, and 2023‐02‐22_P1_02. mtOM, mitochondrial outer membrane.

RP1 and RP2 contain a test for the integrity of the mtOM. The small haem protein cytochrome *c* provides the redox link between CIII and CIV. Ten micromolar cytochrome *c* is kinetically saturating for the S‐pathway (Gnaiger & Kuznetsov, 2002). Stimulation of respiration by cytochrome *c* indicates either artificial damage to the mtOM during sample preparation or a pathophysiological modification (Figure 12a). The release of cytochrome *c* across the mtOM from the intermembrane space is associated with apoptosis. Cytochrome *c* control efficiency is expressed, at a given coupling and substrate state (CHNO), as the increase of respiratory capacity after addition of cytochrome *c* normalized by *c*‐stimulated respiration (Figure 12b): (*J*_CHNO[c]_ − *J*_CHNO_)/*J*_CHNO[c]_ (Gnaiger, 2020).

Selecting the step of cytochrome *c* addition was of primary importance during optimization of RP1 and RP2 (Figure 12). In the leak state, the addition of cytochrome *c* increased oxygen consumption, independent of mtOM integrity. Different cytochrome *c* effects might be triggered when analysed in different pathways. In RP2, cytochrome *c* titration was compared before and after fatty acid titration following the addition of 0.1 mM malate. The F‐pathway capacity was not affected by the sequence of cytochrome *c* titration (Figure 12c), but the cytochrome *c* effect was more pronounced when adding it after octanoylcarnitine. In RP2, cytochrome *c* in the ren state or in the absence of octanoylcarnitine produced a respirometric artifact (Figure 12d, e), even in the presence of antimycin A (Figure 12f), making interpretation of the cytochrome *c* control efficiency questionable in those states. The addition of cytochrome *c* at an early stage ensures comparability of fluxes in a SUIT protocol.

#### 2.4.6 Data analysis in non‐steady state conditions

**FIGURE 13:**
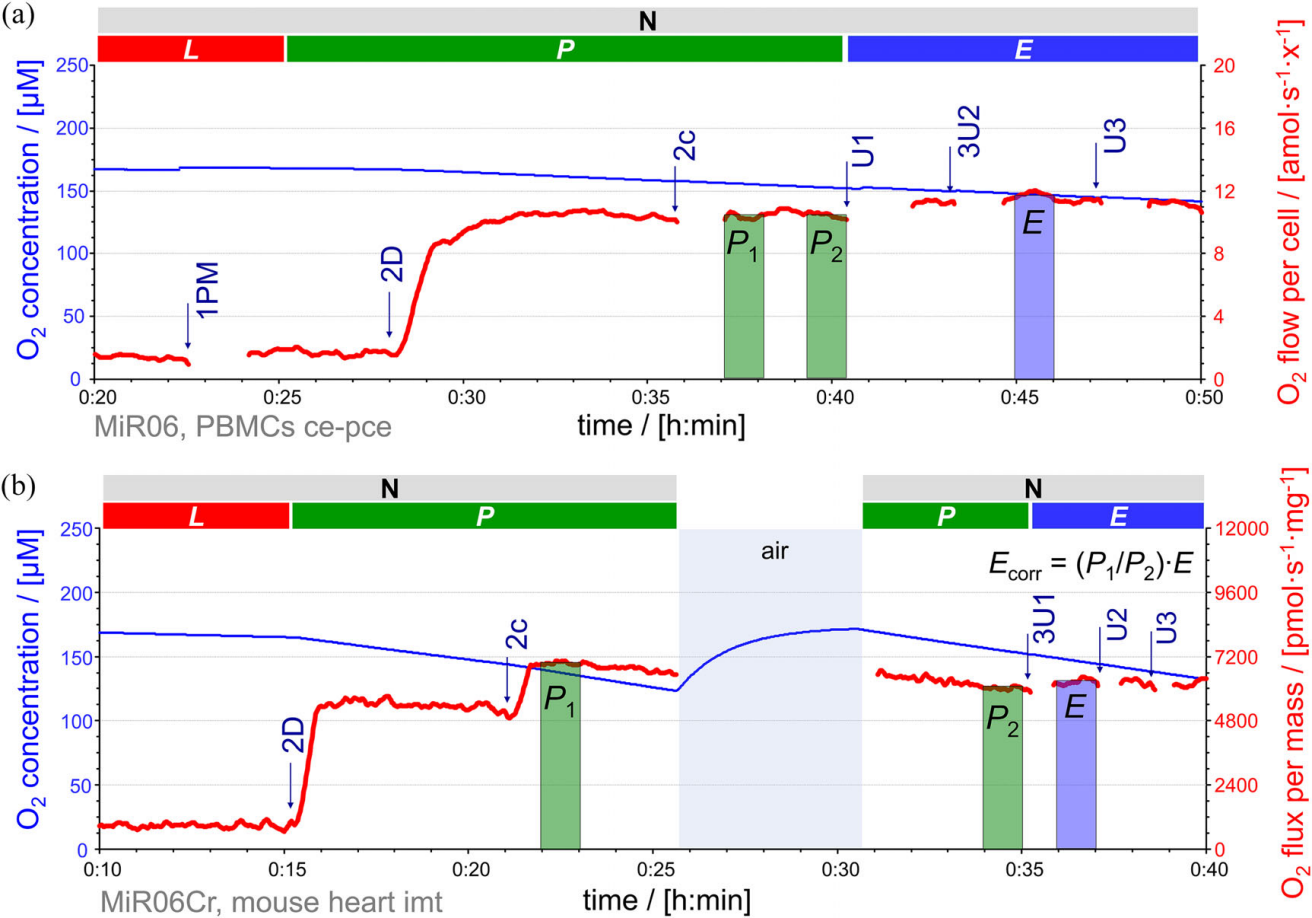
*P*/*E* ratio. Representative traces of sections of RP1 of O_2_ concentration (µM) (blue line) and O_2_ flux per cell (a) or per mitochondrial protein (b) (red lines). Titration spikes were eliminated. Sequence of respiratory states, characterized by titrations and corresponding rates: (a) 1PM; N{PM}*_L_*. 2D; N{PM}*_P_*. 2c, steady state with *P*_1_ = *P*_2_. 3U2, optimum CCCP concentration 2 µM; N{PM}*_E_*. (b) 2D; N{PM}*_P_*. 2c, non‐steady state with *P*_1_ > *E*, but *P*_2_ ≤ *E*. 3U1, optimum CCCP concentration 1 µM; N{PM}*_E_*. Calculation of *E*_corr_ is shown. Air: reoxygenation by opening the chamber. See Table 4 for abbreviations; for further details, see Figure 2 and Table 6. Data repository (Timón‐Gómez et al., 2024): 2016‐10‐05 PS4‐02 and 2017‐02‐06 P2‐02.

Comparison of respiratory rates in different states is based on the assumption that respiratory capacities are exclusively under the control of the titration steps and are otherwise at a steady state over short periods of time. In some cases, however, a time‐dependent change of respiration is observed. If rates are increasing, prolonged monitoring is possible until a steady state is achieved (e.g., after titration of ADP in Figure 2b). In contrast, when respiration declines over time, simply setting a single mark at each respiratory state may yield inconsistent results and requires corrections (Figure 13).

In a step‐analysis, the initial mark defines the background rate *Y*_1_ immediately before a titration. The mark after the titration defines the reference rate *Z*_1_ (Gnaiger, 2020). Then, the next titration step is analysed again by a background rate *Y*_2_ and a reference rate *Z*_2_. This leads to two rates *Z*_1_ and *Y*_2_ in the same respiratory state, which are identical at steady state (Figure 13a, *P*_2_ = *P*_1_). In Figure 13b, *Y*_2_ < *Z*_1_. Therefore, OXPHOS capacities differ, *P*_2_ < *P*_1_. If the *P*/*E* ratio were calculated from *P*_1_/*E* (where *E* = *Z*_2_), we would obtain a value of 1.09, which is theoretically impossible, instead of *P*_2_/*E* = 0.99. The corrected *E*_corr_ (corresponding to *P*_1_) is calculated assuming that the *P*/*E* ratios remain identical for *P*_1_ and *P*_2_:

(1) $$E_{\mathrm{corr}}=\left(P_{1}/P_{2}\right)\times E$$

### 2.5 Statistics

Data was analysed with GraphPad Prism 7 software (GraphPad Software, San Diego, CA, USA). The non‐parametric Kolmogorov–Smirnov test was used to assess the normal distribution of the data. Then, the data were analysed by one‐way ANOVA followed by unpaired Student–Newman–Keuls *post hoc* tests for multiple comparisons with correction. Data is expressed as median ± interquartile range or mean ± standard deviation, indicated in each figure and table. *N* is the number of replica, *n* is the number of technical repeats.

Inverted regression analysis was used to calculate mean inverted regression lines between variables *X* and *Y* (Gnaiger, 2021). Slopes *b_X_* and *b_Y_* and intercepts *a_X_* and *a_Y_* were determined for the *Y*/*X* and *X*/*Y* inverted linear regressions, respectively, to reduce the residuals between both variables. Results are shown as mean slope $\bar{b\;}$ = (*b_Y_* + *b_X_*)/2 and mean intercept $\bar{a}$ = (*a_X_* + *a_Y_*)/2, where *b_X_* = 1/*β_X_* and *a_X_* = −*α_X_*/*β_X_*. Isolinear clusters are represented with a unique inverted regression line and equation in the graph, whereas heterolinear clusters are shown separately to visualize the differences between clusters.

## 3 RESULTS

### 3.1 Comparable states in RP1 and RP2

To gain insights into the mitochondrial function of different tissues, we assessed mitochondrial respiration of mouse brain homogenate, mitochondria isolated from mouse cardiac tissue, permeabilized HEK 293T cells and human blood cells (PBMCs and platelets), using RP1 and RP2. In platelets and PBMCs, octanoylcarnitine exerted an inhibitory effect on N‐ and NS‐pathways (Figure 7). Therefore, a modification of RP1 without octanoylcarnitine was preferred for assessing the respiratory capacity of platelets and PBMCs.

Routine respiration of living cells is measured prior to permeabilization by digitonin in both RP1 and RP2. This is an *identical* respiratory state in both protocols. A second type of comparable state is *harmonized* states with the same pathway‐coupling control conditions which, however, are obtained after different titration sequences in the two SUIT protocols (Figure 2a). Identical states can be used as technical repeats; however, there is no conceptual prediction that rates in harmonized states might be equal because titration sequences might exert effects on the respiratory capacities (Votion et al., 2012).

**FIGURE 14:**
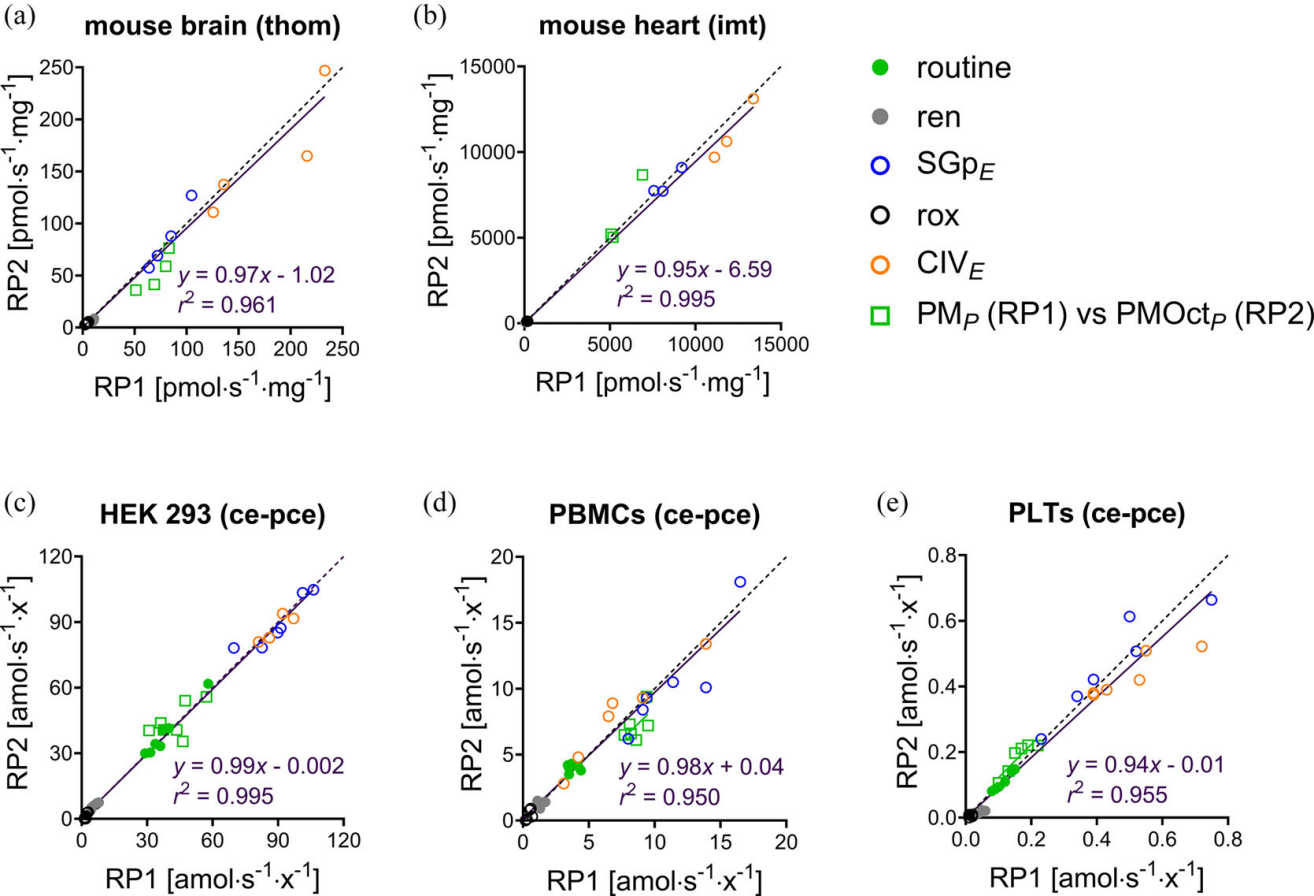
Comparable states between RP1 and RP2 can be used as technical repeats. Inverted regression analysis in (a) brain tissue homogenate from mouse (*N *= 4), (b) cardiac isolated mitochondria from mouse (*N *= 3), and permeabilized (c) HEK 293T cells (*N *= 7), (d) human peripheral blood mononuclear cells PBMCs (*N *= 6), and (e) human platelets PLTs (*N *= 6). Results are expressed as O_2_ flux (pmol s^−1^ mg^−1^) or flow (amol s^−1^ x^−1^), calculated as O_2_ flux per *V* (pmol s^­1^ mL^­1^) divided by concentrations of tissue wet mass (a), protein mass (b) or cell count (c–e) in the Oroboros chamber. Dashed black line: theoretical line of correspondence. Solid purple line and equation: inverted regression analysis for the comparable states (without including possibly comparable states). Solid dots represent identical states: routine (ce1); ren (+Dig); and empty dots represent harmonized states: SGp*_E_* (8Gp in RP1 and 8Rot in RP2); rox (9Ama); PM*_P_* (2D(c) in RP1); PMOct*_P_* (3P in RP2). Empty squares correspond to possibly comparable states (PM*_P_* in RP1 and PMOct*_P_* in RP2, only comparable in the case that Oct does not exert any additive effect, see Section 3.2). Data from Figure 4. See Figure 2 for details of respiratory states in RP1 and RP2.

Comparable states, either identical (routine, when using living cells, and ren) or harmonized (SGp*_E_*, rox and CIV), are represented in RP1 and RP2 (Figure 2a). In these comparable respiratory states, rates were reproducible in the parallel experimental design between RP1 and RP2 in every sample type tested in this work (Figures 4 and 14; representative traces in Figures 2, 5 and 6).

### 3.2 Additive effect of fatty acid oxidation

In agreement with the limited role of FAO in supporting ${\dot{V}}_{O_{2}\max}$ (Achten et al., 2002; Coyle et al., 1997; Horowitz et al., 1997; Romijn et al., 1993), we hypothesized that Oct does not exert an additive effect on the N‐ or NS‐pathway OXPHOS capacity. This was confirmed by the lack of a stimulatory effect of Oct on PGMS*_E_* in RP1 in the models analysed (Figure 4). Zero additivity of FAO was indicated by cluster analysis and the direct proportionality between PM*_P_* in RP1 and PMOct*_P_* in RP2 (Figure 14).

### 3.3 Comparison of OXPHOS and ET capacities

**FIGURE 15:**
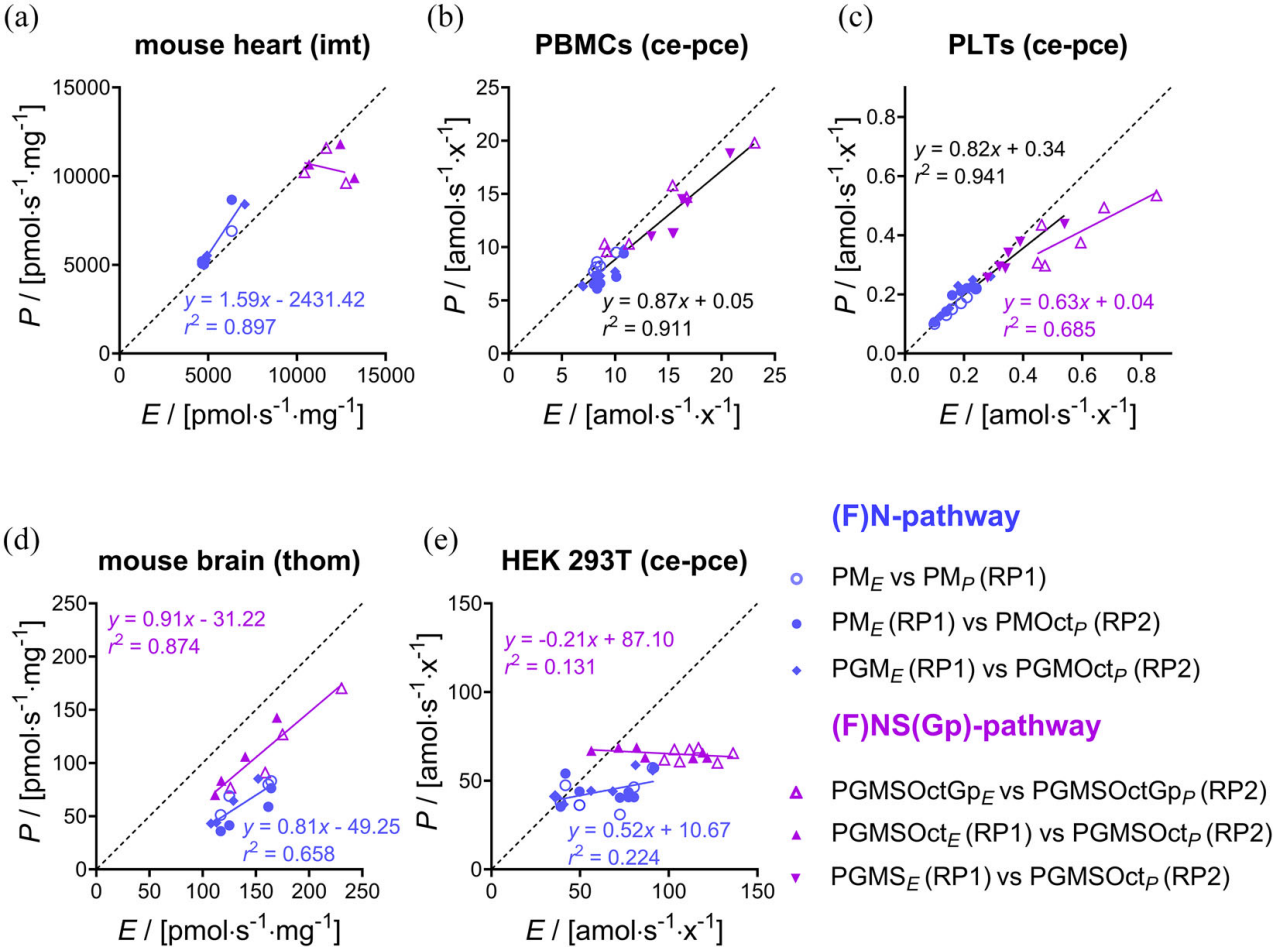
Correlation between fluxes *P* and *E* in OXPHOS and ET states. Inverted regression analysis (a) imt from mouse heart (*N *= 3), (b) pce human PBMCs (*N *= 6), (c) pce human platelets (*N *= 6), (d) thom mouse brain (*N *= 4), (e) pce HEK 293T cells (*N *= 7). O_2_ flux (pmol s^−1^ mg^−1^) or flow (amol s^−1^ x^−1^) calculated as O_2_ flux per *V* (pmol s^−1^ mL^−1^) divided by concentrations of protein mass (imt), tissue wet mass (thom), or cell count (pce) in the Oroboros chamber. Dashed black lines: theoretical line of correspondence. Continuous black lines and equations: inverted regression analysis of isolinear clusters. Heterolinear clusters appear when the phosphorylation system exerts any limitation, as in mouse brain and HEK 293T cells; and the regression analysis (line and equation) is separated into (F)NS(Gp) and (F)N pathways in purple and blue, respectively. States in RP1: PM*_P_*, 2D(c); PM*_E_*, 3U; PGM*_E_*, 4G; PGMS*_E_*, 5S; PGMSOct*_E_*, 6Oct. States in RP2: PMOct*_P_*, 3P; PGMOct*_P_*, 4G; PGMSOct*_P_*, 5S; PGMSOctGp*_P_*, 6Gp; PGMSOctGp*_E_*, 7U. See Figure 2 for respiratory states in RP1 and RP2 and Tables 3 and 4 for abbreviations. Data from Figure 4; see Table 5.

The uncoupler titrations – shifting the coupling state from OXPHOS to ET – were performed in RP1 and RP2 in the N{PM}‐ and FN{PGM}SGp‐pathway states, respectively. These allow for calculation of intra‐assay *E* ‐ *P* control efficiencies. In contrast, an inter‐assay *E* ‐ *P* control efficiency is obtained in the FNS state when performing RP1 and RP2 in parallel (FNS*_P_*/FNS*_E_*; analysed in Figure 15a,d,e). In blood cells (Figure 15b,c), Oct was not added in RP1. Nevertheless, the relation FNS*_P_*/NS*_E_* was used to calculate the *E* ‐ *P* control efficiency since Oct did not stimulate NS*_E_* (Figure 14). Similarly, considering the lack of an additive effect of Oct on the N‐pathway capacity, the relations FN*_P_* / N*_E_* from RP2 and RP1, respectively, was compared by cluster analysis (Figure 15).

Identical respiratory rates in the OXPHOS and ET states (*P* = *E*) were obtained in heart and PBMC mitochondria. These samples had *E ‐ P* control efficiencies (normalized *E ‐ P* excess capacity) of approximately 0, not only in the N‐pathway at low *E* but also in the FNSGp‐pathway with about double *E* (Table 5). This indicates a high phosphorylation capacity for utilizing the protonmotive force that is matched even with the high ET capacity of convergent pathways generating the protonmotive force. A trend may be seen, however, of a slight increase in the *E* ‐ *P* control efficiency toward the highest *E* (Figure 15a,b). Whereas the normalized *E* ‐ *P* excess capacity was close to 0 in the N‐pathway in platelets, it increased to 0.2 in the FNSGp state when *E* doubled at an NS ‐ N control efficiency of 0.5 (Table 5). *P* could cope with the lower *E* in the N‐pathway but was driven to saturation as the ET capacity increased with the FNSGp substrate combination (Figure 15c).

**TABLE 5:** *E* ‐ *P* control efficiency in RP1 and RP2.

Species	Tissue/cell type	Prep. (*N*)	*E* ‐ *P* control efficiency (*E* − *P*)/*E* in N‐pathway^*^	*E* ‐ *P* control efficiency (*E* − *P*)/*E* in (F)NS(Gp)‐pathway^**^
Mouse	Heart	imt (3)	0.08 ± 0.06	0.09 ± 0.11
Human	PBMC	pce (6)	0.12 ± 0.12	0.10 ± 0.14
Human	Platelet	pce (6)	0.05 ± 0.14	0.19 ± 0.15
Mouse	Brain	thom (4)	0.56 ± 0.14	0.34 ± 0.13
Human	HEK 293T	pce (7)	0.26 ± 0.21	0.33 ± 0.20

*Note*: Data (from Figure 4) are means ± SD. See Tables 1, 3 and 4 for abbreviations.

* (F)N‐pathway *E* ‐ *P* control efficiency calculated from the ratios N{PM}*_P_* (RP1)/N{PM}*_E_* (RP1) and FN{PM}*_P_* (RP2)/N{PM}*_E_* (RP1) where FN{PM}*_P_* is referred to as PMOct*_P_*; this yields the *P*/*E* ratio simply indicated as FN*_P_* (RP2)/N*_E_* (RP1).

** (F)NS(Gp) pathway *E ‐P* control efficiency calculated from the ratios FNSGp*_P_* (RP2)/FNSGp*_E_* (RP2) and FNS*_P_* (RP2)/(F)NS*_E_* (RP1).

In brain mitochondria, *P* was lower than the corresponding *E* in the N‐pathway, which means that the phosphorylation system limited OXPHOS capacity. Respiration was increased more than 2‐fold in the transition from OXPHOS to ET, and therefore, the *E ‐ P* excess capacity was as high as 0.56 (Table 5). Compared to the trend observed in the platelets of an increasing *E ‐ P* excess capacity with an increase of *E* from the N‐ to the NS(Gp)‐pathway (Figure 15c), it may be surprising to see an increase of *P* relative to *E* in the NS(Gp)‐pathway (Figure 15d) or even a decrease of the *E ‐ P* excess capacity from 0.56 to 0.34 (Table 5). Two features of respiratory control provide an explanation. (1) At a low additivity between the N‐ and S‐pathways in the ET state in brain (Table 8), there is no further increase in the limitation by the phosphorylation system. Therefore, a constant rather than increased *E ‐* *P* excess capacity can be explained. (2) A decreased *E* ‐ *P* excess capacity requires consideration of substrate competition (Haslam & Krebs, 1963), such that addition of succinate may partially outcompete the contribution of the N‐pathway in the combined NS‐pathway state. Under these conditions, there is a shift from three coupling sites in the N‐pathway to two coupling sites in the S‐ or Gp‐pathways. For the same amount of O_2_ consumed, fewer H^+^ are pumped across the mitochondrial membrane when two rather than three coupling sites are involved (Cardoso & Gnaiger, 2024). Even at the same rate of ATP production, oxygen consumption increases from the N‐ to the NS‐pathway, which becomes apparent as an increased respiratory OXPHOS capacity and subsequent decrease in *E* ‐ *P* control efficiency. This is seen in Figure 15d as two separate heterolinear clusters.

Different batches of cryopreserved HEK 293T cells had variable ET capacity at constant OXPHOS capacity. Figure 15e shows two separate clusters which indicate an increased *E* ‐ *P* control efficiency at the higher FNSGp flow. This pattern explains the large variability of the *E* ‐ *P* control efficiencies (Table 5). The numerical analysis of *E* ‐ *P* control efficiencies masks the separation of distinct clusters, and therefore, it does not disclose the dependence of *E* ‐ *P* control efficiency on NS‐pathway flux as shown by cluster analysis (Table 5 and Figure 15e).

### 3.4 Coupling control

**FIGURE 16:**
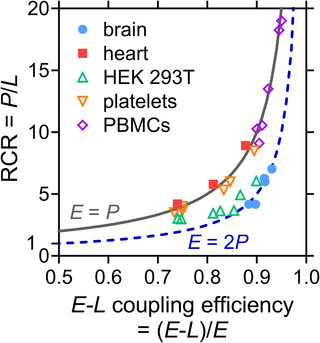
Respiratory coupling coefficients. Acceptor control ratio RCR (theoretical range 1 to infinity) transformed to biochemical *E ‐ L* coupling efficiency = (*E* ‐ *L*)/*E* (theoretical range 0 to 1) from mouse heart imt (*N *= 3) and mouse brain thom (*N *= 4) (closed symbols), HEK 293T cells (*N *= 7), human PBMCs (*N *= 6), and platelets (*N *= 6) (open symbols). The high *E ‐ L* coupling efficiencies of PBMCs had a low variability, whereas the extremely high scatter of corresponding RCR values is not statistically representative of variability of the properly transformed data. Data from Figure 4. Theoretical values of *E* = *P* and *E* = 2*P* are shown as black and blue dashed lines, respectively. See Table 3 for abbreviations.

The classical respiratory acceptor control ratio (RCR) has been considered as an index of coupling control (Chance & Williams, 1955). However, even if OXPHOS is measured at kinetically saturating concentrations of ADP and inorganic phosphate, the corresponding *P*/*L* ratio is under kinetic control of the phosphorylation system when the *E ‐ P* control efficiency is >0 (Table 5). To eliminate the confounding effect of kinetic control from coupling control, the RCR (*P*/*L* ratio) is replaced by the *E*/*L* ratio (Cardoso & Gnaiger, 2024). Inverting the *E*/*L* ratio (ranging from 1 to infinity) to the *L*/*E* ratio is effectively a linearization to the range of 1 to 0, which is required for statistical analysis. Instead of *L*/*E*, the biochemical *E ‐ L* coupling efficiency, 1 − *L*/*E* = (*E* − *L*)/*E*, is used, which increases with increasing RCR (Gnaiger, 2020). When *P *= *E*, the biochemical *E ‐ L* coupling efficiency is equal to the *P‐L* control efficiency, defined as (*P − L*)/*P*. Under completely dyscoupled or uncoupled conditions, biochemical *E ‐ L* coupling efficiency is equal to 0 while RCR equals 1. In a fully coupled system, biochemical *E ‐ L* coupling efficiency increases to its maximum value of 1, whereas RCR approaches infinity (Figure 16).

Coupling control indices in the N‐pathway (RP1) are compared in Tables 6 and 7. The *P*/*L* ratio (RCR) was more than double in PBMCs (12.4) compared to platelets (4.7), whereas the corresponding *P‐L* control efficiency merely decreased from 0.92 to 0.79. The *P‐L* control efficiency of 0.81 in brain homogenate was similar to that of platelets (0.79), whereas the high biochemical *E ‐ L* coupling efficiency of the brain (0.91) was comparable to 0.92 in PBMCs (Figure 16). A limitation of these ratios is that changing leak respiration or ET capacity cannot be distinguished. Consideration of the individual terms is necessary for correct interpretation of the data (Cardoso & Gnaiger, 2024).

**TABLE 6:** Coupling control ratios in the N{PM} pathway.

Species	Tissue/ cell type	Prep. (*N*)	^*^ *P*/*L* Respiratory control ratio RCR	(*P* − *L*)/*P*	^*^ *E*/*L*	(*E − L*)/*E* Biochemical coupling efficiency
Mouse	Heart	imt (3)	6.3	0.83 ± 0.03	6.4^**^	0.81 ± 0.07
Human	PBMC	pce (6)	13.4	0.92 ± 0.02	13.8	0.92 ± 0.02
Human	Platelet	pce (6)	5.1	0.79 ± 0.07	5.6	0.80 ± 0.07
Mouse	Brain	thom (4)	5.5	0.81 ± 0.05	11.3	0.91 ± 0.02
Human	HEK 293T	pce (7)	3.9	0.73 ± 0.03	6.2	0.82 ± 0.02

*Note*: Data from Figure 4 shown as means ± SD. See Table 1 for abbreviations.

* Calculated as the average of the *P*/*L* or *E*/*L* ratios (statistically incorrect; see Table 7).

** *E*_corr_/*L* (see Equation 1 and Figure 13).

**TABLE 7:** Respiratory control ratio (*P*/*L*), its linearization (*L*/*P*) and *P*‐*L* control efficiency.

Species	Tissue/ cell type	Prep. (*N*)	*L*/*P* ^*^	*P*/*L = * 1/(*L*/*P*)^**^	*P*/*L* ^***^	(*P*/*L*)_range_	*P‐L* control eff., 1 − *L*/*P*	(*P‐L* control eff.)_range_
Mouse	Heart	imt (3)	0.18 ± 0.06	5.7	6.3	3.9–8.7	0.82	0.76–0.89
Human	PBMC	pce (6)	0.08 ± 0.02	12.4	13.4	9.2–17.7	0.92	0.90–0.95
Human	Platelet	pce (6)	0.21 ± 0.07	4.7	5.1	3.3–7.1	0.79	0.71–0.83
Mouse	Brain	thom (4)	0.19 ± 0.05	5.3	5.5	4.2–6.8	0.81	0.76–0.86
Human	HEK 293T	pce (7)	0.27 ± 0.06	3.7	3.9	2.8–5.1	0.73	0.66–0.83

*Note*: Data from Figure 4 shown as means ± SD. See Table 1 for abbreviations.

* The linearization from *P*/*L* to *L*/*P* ratio: $\frac{1}{n}\sum_{i=1}^{n}\left(\frac{L}{P}\right)$. It allows the statistical use of mean ± standard deviation.

** Correct calculation of RCR: ${\left(\frac{1}{n}\sum_{i=1}^{n}\frac{L}{P}\right)}^{-1}.$

*** Calculated as: $\frac{1}{n}\sum_{i=1}^{n}\left(\frac{P}{L}\right).$ Statistically not correct but frequently used in the literature for calculation of the RCR.

### 3.5 Additivity of NADH‐ and succinate‐linked ET

The degree of additivity of convergent electron flow into the Q‐junction relates the capacity of the combined NS‐pathway to the arithmetic sum of the separate N‐ and S‐pathways. RP1 addresses the additivity between N‐ and S‐pathways in the ET state (steps 4, 5 and 7, Figure 2a), avoiding any limitation by the phosphorylation system. The pathway control ratios of fluxes through the convergent pathways are indicated as *α* for the predominant pathway (either N or S), and *β* for the subdominant pathway normalized for NS*_E_* (Table 8). Complete additivity of the N‐ and S‐pathways is obtained when the capacity measured with combined substrates is identical to the arithmetic sum of separately measured respiratory capacities (NS = N + S); then *α* + *β* = 1. However, in most cases, additivity was incomplete (NS < N + S). The additivity index is calculated as: $A_{\overset{ˇ}{\alpha \beta }}$  = (1 − *α*)/*β*, which equals 1 at complete additivity. Zero additivity indicates that the combined pathway capacity equals the capacity of the *α*‐pathway, such that the additional substrate (*β*‐pathway) does not exert any stimulatory effect (Gnaiger, 2020).

**TABLE 8:** Additivity of convergent NS‐electron flow in the ET state in mouse and human models.

Species	Tissue or cell type	Prep. (*N*)	N/S	N/$\overset{ˇ}{\mathrm{NS}}$ pathway control ratio	S/$\overset{ˇ}{\mathrm{NS}}$ pathway control ratio	Additivity $A_{\overset{ˇ}{\mathrm{NS}}}$
Mouse	Heart	imt (3)	0.67 ± 0.18	0.45 ± 0.07 (*β*)	0.69 ± 0.08 (*α*)	0.79 ± 0.03
Human	PBMC	pce (6)	0.75 ± 0.21	0.52 ± 0.10 (*β*)	0.70 ± 0.07 (*α*)	0.68 ± 0.08
Human	Platelet	pce (6)	0.79 ± 0.24	0.52 ± 0.12 (*β*)	0.68 ± 0.09 (*α*)	0.71 ± 0.13
Mouse	Brain	thom (4)	2.57 ± 0.13	0.86 ± 0.04 (*α*)	0.33 ± 0.01(*β*)	0.43 ± 0.13
Human	HEK 293T	pce (7)	0.80 ± 0.16	0.62 ± 0.14 (*β*)	0.78 ± 0.06 (*α*)	0.49 ± 0.18

*Note*: The dominant N‐ or S‐pathway is indicated as (*α*). In most cases, the N‐pathway is subdominant (*β*). Data (from Figure 4) are means ± SD. See Table 1 for abbreviations.

The S‐pathway was the dominant *α*‐pathway except for mouse brain, where the N‐pathway capacity was 2.5 times higher than the S‐pathway capacity. The lowest additivities of 0.4 and 0.5 were observed in brain and HEK 293T cells, respectively, whereas additivities were 0.7 and 0.8 in blood cells and heart. The possible correlation between low *E ‐ P* control efficiency (Table 5) and high additivity (Table 8) requires further investigation. These differences in additivity illustrate the diversity of bioenergetic profiles in comparative mitochondrial physiology.

Incomplete additivity (Table 8; Gnaiger, 2020; Komlódi et al., 2021) provides an argument against complete channelling through supercomplex CI–III–IV. This disputes the existence of two tightly separated coenzyme Q‐pools: one Q‐pool within supercomplexes compartmentalizing the ET from NADH (N‐pathway), whereas succinate oxidation (S‐pathway) would proceed separately using a free Q‐pool. In the case of completely separated Q‐pools, the sum of separately measured N and S fluxes would be equal to the NS flux (complete additivity), in contrast to our data (NS < N+S; Table 8). Incomplete additivity ($A_{\overset{ˇ}{\alpha \beta }}$ < 1) implies that the sum of upstream capacities of ET through the N‐ and S‐branches into the Q‐junction is higher than the ET capacity downstream of Q (Figure 1). Our results are in agreement with previous studies (Blaza et al., 2014; Fedor & Hirst, 2018; Letts et al., 2016), although controversies remain in the topic of compartmentalization (reviewed in Hernansanz‐Agustín & Enríquez, 2021). Complete additivity ($A_{\overset{ˇ}{\alpha \beta }}$ = 1) is observed in some cell types and tissues (Hernansanz‐Agustín et al., 2020; Nesci et al., 2023).

### 3.6 Bioenergetic profiles

**FIGURE 17:**
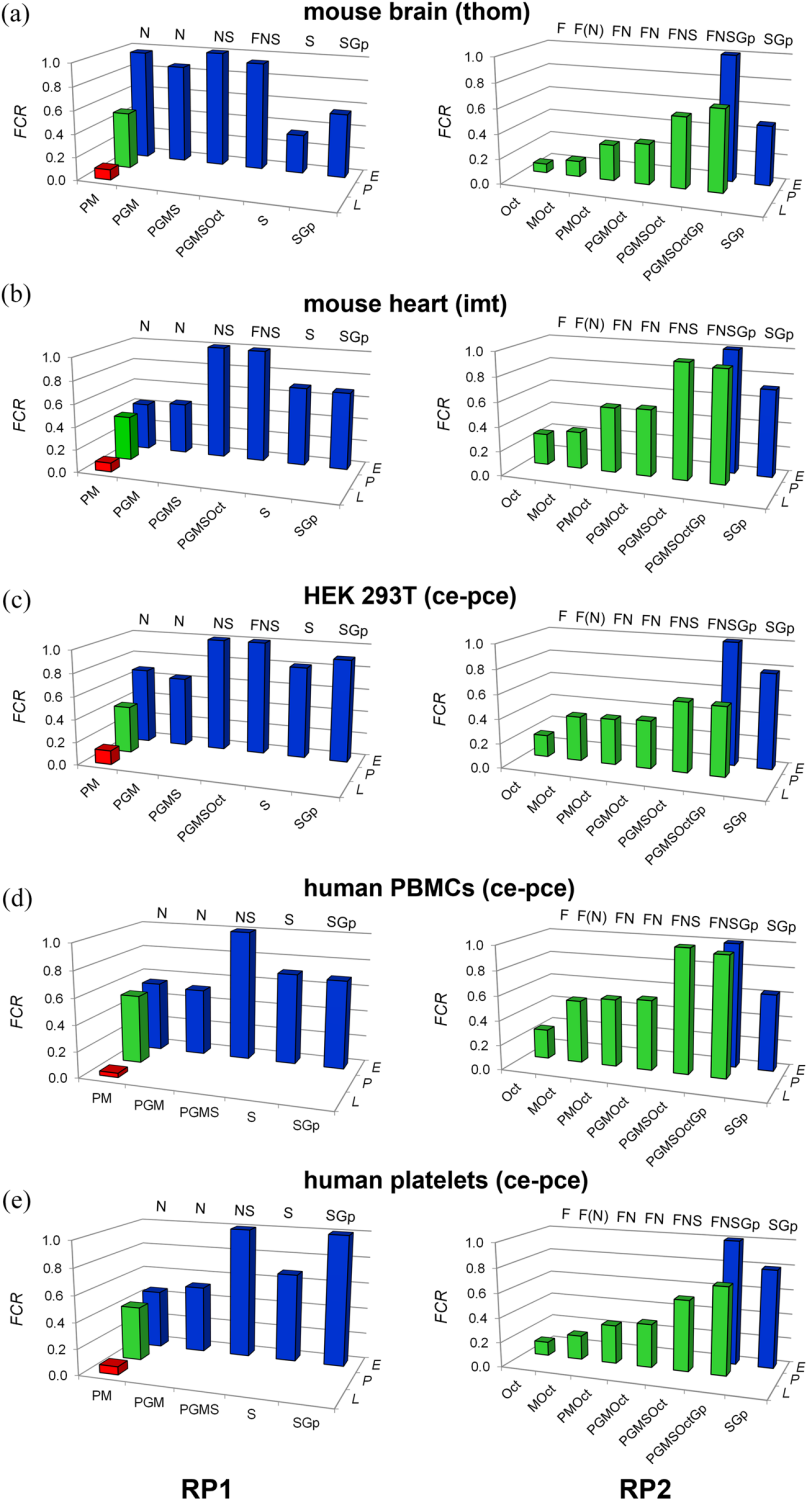
Bioenergetic OXPHOS profiles for comparison of pathway and coupling control in mitochondria of murine tissues and human cell types. Flux control ratios (*FCR*) are normalized by a common reference rate, NS*_E_* in RP1 and FNSGp*_E_* in RP2. Results are shown as the median of *FCR*. Mouse brain thom, *N *= 4; mouse heart imt, *N *= 3; human HEK 293T, *N *= 7; human PBMCs, *N *= 6; human platelets, *N *= 6. Data from Figure 4 (Timón‐Gómez et al., 2024). Representative traces in Figure 2 (HEK 293T), Figure 5 (mouse heart and brain) and Figure 6 (human PBMCs and platelets). See Figure 2 for respiratory states.

Precision OXPHOS analysis with RP1 and RP2 provides bioenergetic profiles in multiple states of ET pathways and coupling control (Figure 17). Data are represented as flux control ratios (*FCR*), which normalize oxygen fluxes for the maximum flux in a common reference state, independent of mitochondrial content and, thus, independent of measuring mitochondrial or cellular concentrations for normalization of flux. Importantly, respiration of different mitochondrial preparations cannot be compared when normalized for preparation‐specific parameters (wet tissue mass, mitochondrial protein content and cell number; Figure 4) or expressed without sample‐specific normalization of flux per volume of the experimental chamber (Figure 2b).

N‐linked oxygen flux was higher compared to the S‐pathway only in mouse brain (Table 8), and both RP1 and RP2 demonstrated a high respiratory control by the phosphorylation system on OXPHOS capacity in this tissue (Figure 17). The F‐pathway was more pronounced in mouse heart mitochondria and PBMCs, compared to the other mitochondria. Mouse brain and platelets showed the highest stimulatory effect of glycerophosphate in the transition from the S‐ to the SGp‐pathway, with a slightly lower effect in HEK 293T cells. The SGp‐pathway capacity matched the NS‐pathway capacity in platelets, but the combined FNSGp‐pathway capacity was higher (Figure 4e). RP2 but not RP1 revealed a high *E* ‐ *P* control efficiency in platelets.

The bioenergetic profile of HEK 293T cells, commonly used as a model to study mitochondrial function, differed from the other tissues and cell types: OXPHOS capacity in HEK 293T cells was limited by the phosphorylation system both in the N‐ and in the FNSGp‐pathway, different from mouse heart and human PBMCs and platelets. However, the S‐pathway was dominant as in the other mitochondria, in contrast to the dominant N‐pathway in mouse brain.

Interestingly, the blood cells, PBMCs and platelets presented strong differences in their mitochondrial respiratory control patterns. PBMCs had low *E ‐ P* excess capacity, in agreement with Calabria et al. (2023), Hsiao & Hoppel (2018) and Theall et al. (2021), but in contrast to results reported by Jang et al. (2018). PBMCs were not stimulated by glycerophosphate, whereas platelets showed a high excess ET capacity with strong stimulation of oxygen flux by the Gp‐pathway. The F‐pathway capacity contributed to a higher extent to the metabolism of PBMCs than platelets. Bioenergetic profiles are cell‐ and tissue‐specific.

## 4 DISCUSSION

Our knowledge of mitochondrial physiology has benefited from respirometric studies with living cells, applying a simple coupling‐control protocol (Hütter et al., 2004; Zdrazilova et al., 2022; Zhang et al., 2012). In this protocol, routine respiration is measured first, which is under physiological control of ATP turnover with OXPHOS operating in parallel to glycolytic ATP production. Intermediary metabolism supplies complex substrate combinations supporting multiple mitochondrial pathways in living cells which cannot be directly controlled. In mitochondrial preparations, on the other hand, pathway control states can be defined and modified by external addition of different substrates and specific inhibitors. Therefore, mitochondrial (dys)function may be assessed on two levels: (1) in a simple coupling‐control protocol with living cells; and (2) by precision OXPHOS analysis with mitochondrial preparations to examine specific bioenergetic profiles (Doerrier et al., 2018; Gnaiger, 2020; Hsiao & Hoppel, 2018) and to characterize specific defects or adaptions (e.g., Baglivo et al., 2024; Leo et al., 2025; Li et al., 2024; Mahapatra et al., 2024). SUIT protocols do not address a single pathway only but interrogate a broad spectrum of OXPHOS function, from routine respiration in living cells to permeabilization of the plasma membrane, to assess metabolic fluxes in defined coupling and pathway control states (Figures 1 and 2).

Mitochondrial preparations have specific advantages and disadvantages: in permeabilized cells and tissues, mitochondria are not fragmented by mechanical homogenization, and all mitochondrial populations are represented (Picard et al., 2011). Isolation of mitochondria requires precautions to preserve integrity, partial loss of which may be related to the use of nagarse (Lai et al., 2019). Substrate‐specific respiration of isolated mitochondria or permeabilized muscle fibres can be validated in relation to whole‐body physiology (Boushel et al., 2015; Lanza et al., 2011; Larsen et al., 2011; Layec et al., 2016; Newsom et al., 2021; Pesta et al., 2011; Smith et al., 2012). Preparation of isolated mitochondria requires centrifugation steps for removal of plasma membranes and other organelles, and a possible selection of mitochondrial subpopulations. Tissue homogenate preparation, instead, is faster and requires less tissue and is possible without the use of detergents (e.g., digitonin). Bioenergetic profiles (Gnaiger, 2020) provide a powerful tool for comparison of results obtained with isolated mitochondria and other mitochondrial preparations (Gnaiger, 2009; Margulies et al., 2015; Perry et al., 2013).

ET pathways have been assessed separately by using either NADH‐linked substrates (CI‐linked N‐pathway) or a succinate + rotenone combination (CII‐linked S‐pathway) (Chance & Williams, 1955). Tissue‐specific patterns have been described using substrates supporting a single pathway (Tomar et al., 2022), but these traditional protocols lead to an underestimation of ET capacity compared to the convergent NS‐pathway, which is physiologically more relevant (Gnaiger, 2020; 2009; Hatefi et al., 1962; Rasmussen et al., 2001). The single pathway analysis leads to an overestimation of CIV excess capacity (Rossignol et al., 2003; Villani & Attardi, 1997; Villani et al., 1998). Restriction to pyruvate + malate as N‐pathway substrates fails to reveal the actual extent of mitochondrial functional diversity in muscle tissues of different species (Hulbert et al., 2006), compared to studies considering the combination of N‐ and S‐pathways in this tissue (Boël et al., 2020; Robinson et al., 2019; Teulier et al., 2019).

Here we describe two SUIT reference protocols, RP1 and RP2, that assess 20 mitochondrial ET pathway and coupling control states (Figures 2, 4, 5, 6). F‐, N‐, S‐, and Gp‐pathways converging at the Q‐junction are assessed either separately or in physiological combinations. Specific enzymatic activity is measured as CIV‐linked respiration. Precision OXPHOS analysis using RP1 and RP2 provides high diagnostic power, linking alterations of respiratory function to molecular components of the ETS.

The additive effect of the F‐pathway might be overestimated when using the N‐pathway as a reference (Davidson et al., 2020; Pedersen et al., 2022) and should be related to NS‐pathway capacity (RP1). After the FNS combination, addition of rotenone allows determination of the S‐pathway capacity since the F‐pathway is inhibited indirectly by rotenone (see limitations of this approach below). The design of RP1 allows calculation of the additivity of the N‐ and S‐pathway capacities in the ET state. In many types of mitochondria, the additivity of convergent pathways in the OXPHOS state is incomplete, which means that the combination of components leads to a lower value than their linear sum (Gnaiger, 2020). In our study, additivity of convergent NS‐ET capacity was incomplete and tissue‐dependent (Table 8).

RP2 was designed to analyse the F‐pathway in the OXPHOS state. FAO reduces ETF, which is the substrate of CETFDH. Simultaneously, NAD^+^ is reduced to NADH in the β‐oxidation cycle, and in the TCA cycle fed by acetyl‐CoA (Gnaiger, 2024; Houten & Wanders, 2010). Therefore, it is incorrect when authors refer to FAO as CII respiration (Tonnesen et al., 2021; for a review see Gnaiger, 2024). The use of a high concentration of malate together with octanoylcarnitine in tissues like kidney (Zhang et al., 2021), which express the mitochondrial malic enzyme (Nagel et al., 1980), requires critical assessment of a possible overestimation of the FAO capacity. Inverting the sequence of titrations, with acylcarnitine before low malate (Panov et al., 2022), prevents the subtraction of the flux after low malate from the flux after acylcarnitine, leading to a possible overestimation of F‐pathway capacity. Other studies report the addition of ADP after octanoylcarnitine (Hoene et al., 2021; McKenna et al., 2021; Palacka et al., 2021), which ignores the possible effect of ADP on flux at low malate concentrations.

Octanoylcarnitine is a substrate used to study FAO in RP2 while avoiding the rate‐limiting CPT1‐mediated transfer step, although other substrates, such as palmitoylcarnitine, might be optimal depending on the model, such as mouse brain homogenate (Cardoso et al., 2025) or PBMCs (Hsiao & Hoppel, 2018). The fatty acids (or acylcarnitines), their combinations and concentrations are critical for measuring the actual F‐pathway capacity compared to simply supplying octanoylcarnitine. Different acylcarnitines may support higher FAO (Cardoso et al., 2025) and exert additive effects, as described for octanoylcarnitine and palmitoylcarnitine in human heart (Lemieux et al., 2011).

Agreement between fluxes in identical and harmonized states in RP1 and RP2 supports a statistical design of experiments in which the two protocols serve as technical repeats when performed in parallel. These comparable states can be used as a reference for reproducibility (Figures 4 and 14).

The leak state may be induced by oligomycin after the combination of NS substrates (Krajčová et al., 2020; Sjövall et al., 2013; Westerlund et al., 2024) or NSGp substrates (Pecina et al., 2014). However, this approach may lead to potential misinterpretation of *E*/*L* or *P*/*L* ratios as indices of coupling control because substrate competition may induce a shift in the contribution of the N‐ and S‐pathway. Similarly, the contributing mitochondrial pathways may change in living cells simultaneously with coupling control when inducing the leak state with oligomycin to inhibit ATP synthase, followed by uncoupler titrations to reach ET capacity. Therefore, interpretation in terms of coupling efficiency may imply an oversimplification, considering that different mitochondrial pathways have different H^+^/O_2_ ratios, which exert an influence on the biochemical *E ‐ L* coupling efficiency. To avoid this complexity in the measurement of coupling efficiency, pathway states are strictly controlled in precision OXPHOS analysis with mitochondrial preparations. It is possible to measure leak rates in specific ET pathways avoiding substrate competition (Gnaiger et al., 2015). Oligomycin addition after CCCP (Babylon et al., 2022; Dieter et al., 2022) does not induce the leak state but is a test of off‐target inhibition of ET capacity by oligomycin.

Although RP1 and RP2 address the contribution of multiple ET pathways and coupling control states, these SUIT protocols present some limitations. One limitation is the lack of evaluation of the contribution of additional dehydrogenases that feed electrons into the Q‐junction (e.g., proline dehydrogenase). Another limitation is that neither RP1 nor RP2 measures the Gp‐pathway capacity alone. The use of malonate, a competitive inhibitor of CII, may be considered to overcome this limitation (Sumbalová et al., 2023). Another limitation of RP1 is the variable inhibition of S‐pathway capacity by malate. Furthermore, not only in RP1 and RP2 but in any SUIT protocol analysing CIV activity, TMPD is not added at saturating concentrations due to its high autooxidation. Thus, the chosen TMPD concentration represents a compromise. Although RP1 and RP2 were designed as standard protocols, some modifications are necessary for specific types of cells, tissues and/or organisms, such as blood cells in RP1 (Figure 7). The use of different fatty acids (Cardoso et al., 2025) and concentrations of some reagents (e.g., digitonin or ADP) must be optimized for each specific model and cell concentration. With millimolar kinetically saturating concentrations of ADP, measurement of ADP/O_2_ ratios is not possible, and specifically designed protocols must be applied for this purpose (Gnaiger, 2001; Gnaiger et al., 2000).

It is well recognized that tissue‐specific differences in mitochondrial function are relevant in understanding the tissue‐specific impact and manifestations in mitochondrial diseases (Balmaceda et al., 2024). In this study, we obtained the bioenergetic profiles of five tissue and cellular models from human and mice samples.

Our results on mitochondria isolated from mouse heart confirmed previous results on mouse cardiac fibres. This tissue has no *E ‐ P* excess capacity (Lemieux et al., 2017), in comparison to the high *E ‐ P* excess capacity observed in human heart, in which N‐linked ET capacity is twice as high as OXPHOS capacity (Lemieux et al., 2011). Differences in respiratory control between human cardiac and skeletal muscle mitochondria described previously in terms of RCRs (Park et al., 2014) are even more pronounced when considering the high *E ‐ P* excess capacity in human cardiac mitochondria (Lemieux et al., 2011) in contrast to *E ‐ P* excess capacities, which are low or even close to zero in trained humans (Doerrier et al., 2024; Pesta et al., 2011). Reports on *E *< *P* (e.g., Iuso et al., 2017) must be disregarded as methodologically induced artifacts. It is important to evaluate steady‐state respiratory rates, avoiding experiments in which respiration changes as a function of time in a given respiratory state, particularly after titration of ADP and uncoupler (Figure 13).

The predominant respiratory pathway in mouse brain tissue homogenate was the N‐pathway, as previously observed (Burtscher et al., 2015), whereas the S‐pathway was the predominant pathway in mouse heart, HEK 293T cells, and human PBMCs and platelets. These results might point toward the brain being more sensitive to CI‐linked dysfunction, whereas the other models could be superior for studying CII‐linked alterations. Shifts toward lower CI‐pathway capacity may be compensated for by increasing S‐pathway capacity in cancer tissue (Schöpf et al., 2020).

Growing evidence suggests that mitochondrial respiratory function of blood cells (PBMCs and platelets) might be used as a marker to detect mitochondrial dysfunction and to assess diverse pathophysiological conditions (Alfatni et al., 2020; Bellar et al., 2023; Braganza et al., 2019; Gumpp et al., 2020; Heimler et al., 2022; Hsiao & Hoppel, 2018; Palacka et al., 2021; Sartori et al., 2024; Theall et al., 2021). Although their mitochondrial function has been previously compared (Chacko et al., 2013), many studies are restricted to coupling control protocols with living cells where precision OXPHOS analysis cannot be applied. Some studies have been performed with permeabilized platelets and PBMCs but mostly focused on their comparison to muscle tissue and without extensive discussion of mitochondrial pathway control (Rose et al., 2019; Wanet et al., 2015; Westerlund et al., 2024). Given the comparable environment in the circulating blood, their mitochondrial bioenergetic profiles were expected to be similar. However, using precision OXPHOS analysis, we unravelled important mitochondrial functional differences between PBMCs and platelets, particularly in Gp‐pathway capacity and coupling control (Figure 17d,e). This highlights the relevance of precision OXPHOS analysis, for example, for taking a decision on the preferred cell type for use in pharmacological studies. As an example, metformin is an antidiabetic drug known to target not only CI, but also glycerophosphate dehydrogenase (Pecinova et al., 2017). Platelet respiration was stimulated by glycerophosphate, in contrast to the lack of stimulation observed in PBMCs (Figure 17d,e). Thus, platelets are the model of choice in liquid biopsies to study off‐target effects in the Gp‐pathway caused by metformin.

HEK 293T cells are used as a reference model in many studies; however, their bioenergetic profile was different from any of the cell types/tissues that we analysed. This highlights the importance of comparative mitochondrial physiology to select the proper model for a specific study and to understand potential discrepancies between results obtained in cultured cells and tissues (Schöpf et al., 2016).

Efficiency is a major functional attribute of OXPHOS. Tissue‐specific differences in *E ‐ L* and *E ‐ P* control efficiencies found in our study are relevant in the context of a comparative physiological database and must be considered to select the right model to answer specific research questions. As an example, PBMCs and mouse heart tissue – having a lower limitation of OXPHOS – might present higher sensitivity to a compromise in those efficiencies than the other models analysed, as previously described in mitochondrial deficiency models (Balmaceda et al., 2024). Our results also highlight the use of biochemical *E* ‐ *L* coupling efficiency instead of the respiratory control ratio, for statistical purposes and to avoid kinetic limitation of coupling efficiency (Tables 5 and 7, and Figure 16).

Applications of RP1 and RP2, or shorter versions of these protocols, have unravelled tissue‐specific profiles (Kappler et al., 2019) and specific mitochondrial respiratory defects in human diseases and ageing (Fuertes‐Agudo et al., 2022; Mahapatra et al., 2024; Sumbalová et al., 2023). Precision OXPHOS analysis provides a guide to and evaluation of potential treatments of mitochondria‐linked diseases (Sumbalová et al., 2023; Torres Quesada et al., 2022). Even if several respirometric variables do not show diagnostic significance, in‐depth OXPHOS analyses reveal correlations between particularly responsive respiratory rates or flux ratios and clinical outcomes (Calabria et al., 2023; Meszaros et al., 2022; Scandalis et al., 2023). These reference protocols have been used for quality control of experimental procedures and mitochondrial respiration media (Baglivo et al., 2024; Doerrier et al., 2024; Leo et al., 2025; Torres‐Quesada et al., 2022). Bioenergetic profiles illuminate the underlying mechanisms and functional consequences of metabolic adaptions in health and disease, enhancing the development of novel therapeutic approaches, such as mitochondrial transplantation (Hayashida et al., 2023; Neikirk et al., 2024).

In conclusion, harmonization of nomenclature and standardized experimental protocols are essential for the advancement of comparative mitochondrial physiology (Gnaiger, 2009; Gnaiger et al., 2020). Here we present two SUIT reference protocols for HRR that can be used to create a mitochondrial pathway and coupling control database for different cell types, tissues and species, to establish bioenergetic profiling in comparative mitochondrial physiology. By interrogating many coupling and pathway control states, a large diversity of mitochondrial function is revealed. Differences in bioenergetic profiles can explain tissue‐specific differences in mitochondrial function; detect metabolic deficiencies in human disorders in comparison to simple remodelling under certain environmental or nutritional conditions; and must be considered in any pharmacological study to select the right model for a specific experimental or clinical question. In summary, bioenergetic profiling serves as a powerful approach to unravel the complexities of mitochondrial respiratory control.

## AUTHOR CONTRIBUTIONS

Conceptualization: Carolina Doerrier, Zuzana Sumbalová, and Erich Gnaiger. Formal analysis: Alba Timón‐Gómez, Carolina Doerrier, Zuzana Sumbalová, and Erich Gnaiger. Investigation: Alba Timón‐Gómez, Carolina Doerrier, Zuzana Sumbalová, Luiz F. Garcia‐Souza, and Eleonora Baglivo. Methodology: Alba Timón‐Gómez, Carolina Doerrier, Zuzana Sumbalová, Luiz F. Garcia‐Souza, and Erich Gnaiger. Supervision: Erich Gnaiger; Visualization, Alba Timón‐Gómez, Carolina Doerrier, and Erich Gnaiger. Writing – Original Draft: Alba Timón‐Gómez, Carolina Doerrier, and Erich Gnaiger. Writing – Review & Editing: Alba Timón‐Gómez, Carolina Doerrier, Zuzana Sumbalová, Luiz F. Garcia‐Souza, Eleonora Baglivo, Luiza HD. Cardoso, and Erich Gnaiger. All authors have read and approved the final version of this manuscript and agree to be accountable for all aspects of the work in ensuring that questions related to the accuracy or integrity of any part of the work are appropriately investigated and resolved. All persons designated as authors qualify for authorship, and all those who qualify for authorship are listed.

## CONFLICT OF INTEREST

Alba Timón‐Gómez, Eleonora Baglivo, and Luiza HD Cardoso are employees of Oroboros Instruments. Carolina Doerrier, Luiz F Garcia‐Souza, and Zuzana Sumbalová were employed by Oroboros Instruments during the project. Erich Gnaiger is the founder and CEO of Oroboros Instruments.

## Data Availability

Original files are available Open Access at Zenodo repository: https://doi.org/10.5281/zenodo.13774074
